# Adaptive Compressed Sensing Differential Privacy Federated Learning Based on Orbital Spatiotemporal Characteristics in Space–Air–Ground Networks

**DOI:** 10.3390/s26061874

**Published:** 2026-03-16

**Authors:** Weibang Li, Ling Li, Lidong Zhu

**Affiliations:** 1 College of Computer Science and Artificial Intelligence, Southwest Minzu University, Chengdu 610041, China; 21700142@swun.edu.cn; 2 National Key Laboratory of Wireless Communications, University of Electronic Science and Technology of China, Chengdu 611731, China; zld@uestc.edu.cn

**Keywords:** space-air-ground integrated networks, federated learning, differential privacy, compressed sensing, orbital dynamics, adaptive optimization, reinforcement learning

## Abstract

With the development of 6G communication technology, Space–Air–Ground Integrated Networks (SAGINs) have become critical infrastructure for global intelligent collaborative computing. However, federated learning deployment in SAGINs faces three severe challenges: the high dynamics of satellite orbital motion, node resource heterogeneity, and privacy vulnerabilities in data transmission. This paper proposes an adaptive compressed sensing differential privacy federated learning framework based on orbital spatiotemporal characteristics. First, we design orbital periodicity-driven time-varying sparse sensing matrices that dynamically adjust compression strategies according to satellite orbital positions, achieving intelligent communication efficiency optimization. Second, we propose an orbital predictability-based privacy budget temporal allocation mechanism and perform differential privacy noise injection in the compressed domain, establishing a compression–privacy joint optimization algorithm. Furthermore, we construct an energy–communication–privacy ternary collaborative mechanism that achieves multi-objective dynamic balance through model predictive control. Finally, we design reinforcement learning-based dynamic routing scheduling and hierarchical aggregation strategies to effectively handle the time-varying characteristics of network topology. Simulation experiments demonstrate that compared to existing methods, the proposed approach achieves 3–12% improvement in model accuracy and 30–50% enhancement in communication efficiency while maintaining differential privacy protection with dynamic privacy budget ε∈[0.1,10.0] and compression ratio ρ∈[0.2,0.8]. Unlike static compressed sensing approaches that ignore orbital periodicity, the proposed orbital-driven time-varying sensing matrices reduce reconstruction error by up to 19.4% compared to fixed-matrix baselines, validating the synergistic effectiveness of integrating orbital spatiotemporal characteristics with federated learning in 6G SAGIN deployments. The framework assumes reliable orbital propagation via SGP4/SDP4 models and does not account for Doppler frequency shifts or inter-satellite link handover delays; future extensions include scalability to mega-constellations and integration of quantum-resistant privacy mechanisms.

## 1. Introduction

### 1.1. Background

**Figure 1:**
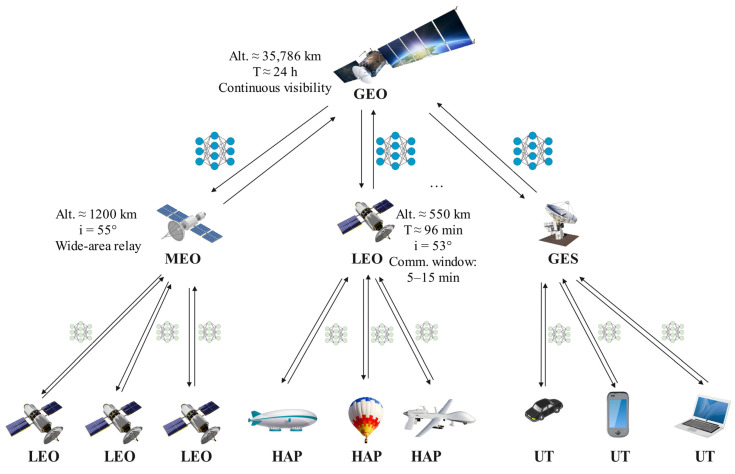
Schematic diagram of three-tier space–air–ground integrated network federated learning architecture. Orbital parameters: GEO (Alt. ≈ 35,786 km, T ≈ 24 h), MEO (Alt. ≈ 1200 km, i = 55°), and LEO (Alt. ≈ 550 km, T ≈ 96 min, i = 53°). Available communication windows: 5–15 min for LEO–GES links, 8–12 min for LEO–HAP links, and continuous visibility for MEO–GEO links. These orbital spatiotemporal characteristics form the basis of the proposed adaptive sensing and privacy allocation mechanisms.

With the deepening advancement of global digital transformation, humanity’s demand for ubiquitous intelligent interconnection services continues to grow. Space–Air–Ground Integrated Networks (SAGINs), as a crucial component of future 6G mobile communication systems, are emerging as key infrastructure for achieving global seamless connectivity and intelligent collaborative computing [1,2]. Particularly with the large-scale deployment of Low Earth Orbit (LEO) satellite constellations by commercial aerospace companies such as SpaceX and OneWeb, the coverage capability and intelligence level of space–air–ground networks have been significantly enhanced [3,4]. As illustrated in Figure 1 (detailed in Section 3), the three-tier SAGIN architecture encompasses GEO satellites at ~35,786 km providing global coverage, MEO satellites at ~1200 km enabling wide-area relay, and LEO satellites at ~550 km with orbital periods of approximately 96 min providing low-latency access to HAPs and ground terminals. The orbital inclinations and Keplerian parameters of LEO/MEO layers introduce predictable but highly dynamic connectivity patterns, making standard federated learning aggregation strategies fundamentally inadequate for such environments.

In this context, Federated Learning (FL), as a privacy-preserving distributed machine learning paradigm, provides novel solutions for intelligent collaboration in space–air–ground networks [5]. Unlike traditional centralized learning, federated learning enables satellite nodes distributed across different orbital layers, High Altitude Platforms (HAPs), and ground terminal devices to achieve collaborative training through model parameter aggregation without sharing raw data [6,7]. This distributed intelligence paradigm demonstrates tremendous potential in space–air–ground network applications such as remote sensing image classification, spatial modulation recognition, and intelligent routing optimization [8,9].

However, federated learning in SAGIN environments faces three unprecedented challenges. First, the periodic orbital motion of satellite nodes causes highly dynamic network topology with time-varying link reachability and quality [10]. Second, the multi-layered heterogeneous architecture introduces significant disparities in computational capability, storage capacity, and energy constraints across node types [11]. Third, data transmitted over SAGINs frequently involves nationally sensitive or commercially confidential information, imposing stringent privacy protection requirements [12].

### 1.2. Motivation and Contributions

The spatiotemporal dynamics of SAGINs are primarily manifested in the periodicity of orbital motion and the time-varying nature of network topology. LEO satellites have orbital periods of 90–120 min, yielding communication windows of only 5–15 min between satellites and ground stations [13]. Inter-satellite link establishment and disconnection exhibit high spatiotemporal correlation, rendering traditional static federated learning aggregation strategies ill-suited for such dynamic environments [14]. As constellation scales continue to expand, achieving efficient model parameter transmission and aggregation within these limited windows becomes a critical performance bottleneck.

The resource heterogeneity of nodes in space–air–ground networks manifests across multiple dimensions. In terms of computational resources, GEO satellites typically possess stronger processing capabilities, while LEO satellites and ground terminal devices have relatively limited computational resources [15]. Regarding energy constraints, satellite nodes primarily rely on solar panels for power supply, and the balance between energy harvesting and consumption directly affects the system’s sustained operational capability [16]. In terms of communication resources, different types of links (inter-satellite links, satellite–ground links, terrestrial links) exhibit significant differences in bandwidth, latency, and reliability [17]. This multi-dimensional resource heterogeneity requires federated learning algorithms to adaptively adjust training strategies and resource allocation schemes.

Although federated learning avoids direct raw data sharing, model parameters can still leak sensitive information. In SAGIN environments, this risk is further amplified by three factors [18]: multi-hop transmission paths increase interception opportunities; orbital predictability enables attackers to infer data characteristics within specific time windows; and complex inter-layer trust relationships expose nodes to gradient inversion and membership inference attacks [19,20].

Facing these challenges, existing federated learning methods primarily suffer from the following limitations: first, lack of deep integration with orbital dynamics characteristics, resulting in algorithm design disconnected from actual deployment environments; second, absence of systematic theoretical frameworks for privacy protection and communication efficiency trade-off optimization; third, neglect of dynamic balancing mechanisms for energy harvesting and consumption in space–air–ground networks.

Specifically, (i) existing approaches addressing communication efficiency in satellite and space networks [16,17] adopt static or fixed transmission strategies that do not account for the periodicity of satellite orbital motion, and even federated learning frameworks incorporating compressed sensing [19,20] employ measurement matrices designed independently of orbital phase dynamics, leading to suboptimal gradient compression performance during unfavorable link conditions; (ii) current privacy-preserving federated learning methods [18,19,20] adopt fixed or heuristic privacy budget allocation schemes without exploiting the temporal predictability of satellite orbits, resulting in inefficient budget utilization—over-consuming privacy resources during high-quality channel windows while providing insufficient protection during vulnerable orbital phases; (iii) the few works that simultaneously consider both compression and privacy [19,20] treat them as independent sequential operations (compress-then-protect or protect-then-compress), failing to exploit the synergistic gains achievable through joint compressed-domain differential privacy design, and none of these works provide rigorous convergence guarantees for the joint optimization.

Based on this analysis, this paper proposes an adaptive privacy-compression cooperative mechanism based on orbital spatiotemporal characteristics as the core innovation motivation. Specifically, our innovation motivation is reflected in the following three aspects:

(1) Orbital-aware spatiotemporal adaptive mechanism: Fully utilizing the predictability of satellite orbital motion to design time-varying compressed sensing matrices and differential privacy budget allocation strategies, enabling algorithms to dynamically adjust optimization objectives according to current orbital positions and communication windows.

(2) Multi-objective cooperative optimization framework: Establishing a theoretical framework for energy–privacy–accuracy tri-element trade-offs, achieving dynamic balance among multiple objectives through Pareto optimal solutions, providing theoretical guidance for space–air–ground federated learning.

(3) Deep integration technical approach: Rather than simple technology stacking, performing differential privacy noise injection within the compressed sensing domain, achieving significant communication load reduction while maintaining privacy protection, creating a synergistic effect where 1 + 1 > 2.

The main contributions of this paper can be summarized in the following three aspects:

(1) Orbital spatiotemporal characteristic-driven adaptive compressed sensing mechanism: For the first time, deeply integrating the periodicity and predictability of satellite orbital motion into compressed sensing design, proposing time-varying sparse sensing matrices based on orbital phases and dynamic compression rate adjustment strategies, achieving intelligent matching between compression strategies and network dynamic characteristics, significantly reducing communication overhead compared to traditional fixed compression methods under the same accuracy requirements.

(2) Differential privacy noise injection and joint optimization within compressed domain: Innovatively performing differential privacy noise injection within the compressed sensing domain, establishing theoretical bounds for global sensitivity after compression, designing orbital-aware privacy budget temporal allocation mechanisms, achieving deep coordination between privacy protection and communication efficiency, effectively improving model accuracy while maintaining the same privacy protection level.

(3) Multi-timescale energy–communication–privacy tri-element cooperative optimization framework: Constructing a tri-element cooperative mechanism based on model predictive control, achieving global optimization of system performance through multi-timescale weight adjustment and reinforcement learning-driven dynamic routing, establishing complete convergence theoretical guarantees, providing systematic theoretical and technical frameworks for space–air–ground federated learning.

Beyond technical contributions, the proposed framework has direct practical implications for global intelligent computing in underserved regions. By enabling efficient and privacy-preserving federated learning within constrained LEO communication windows (5–15 min per pass), the framework supports deployment scenarios including remote environmental monitoring, disaster response coordination, and distributed inference in areas lacking terrestrial network infrastructure—contexts where 6G SAGIN is poised to become the primary connectivity fabric.

## 2. Related Work

### 2.1. Federated Learning in Satellite Networks

The rapid expansion of commercial LEO satellite constellations has opened new opportunities for space-based intelligent computing. Federated learning, as a machine learning paradigm naturally suited for distributed environments, has received widespread attention in satellite network applications [21]. Lin et al. proposed the FedSN framework, systematically addressing heterogeneous federated learning problems in LEO satellite networks for the first time [3]. To address the dynamics of satellite–ground communication links, researchers have proposed various asynchronous aggregation mechanisms. Xu et al. proposed a connection density-aware satellite–ground federated learning framework [22], which achieves dynamic asynchronous aggregation strategies by sensing the connection density between satellites and ground stations, effectively alleviating communication congestion problems caused by synchronous waiting. Recent research has begun focusing on the impact of orbital dynamics characteristics on federated learning performance. Lozano-Cuadra et al. proposed a decentralized satellite routing scheme based on continual deep reinforcement learning [23], which utilizes the predictability of orbital dynamics to achieve continual learning through model prediction and federated learning approaches. However, this work primarily focuses on routing optimization and does not deeply explore privacy protection mechanisms.

### 2.2. Compressed Sensing Applications in Distributed Learning

Compressed sensing has attracted attention in federated learning for its ability to significantly reduce data transmission volume while preserving information integrity. Jeon et al. were the first to integrate compressed sensing into large-scale MIMO federated learning [24], proposing sparse transmission strategies and iterative LMMSE estimation algorithms that substantially reduce communication overhead without sacrificing model accuracy. Experiments showed that this method could significantly reduce communication overhead while maintaining model accuracy. Li et al. proposed the MCFL-CS scheme, combining model contrastive learning with improved compressed sensing [25]. This method alleviates the impact of data heterogeneity on model accuracy by jointly optimizing model contrastive loss and cross-entropy loss, while using compressed sensing and local differential privacy to reduce communication costs and prevent privacy leakage. However, the compressed sensing matrix design in this work is relatively static, not considering the impact of network dynamics. Addressing the problem that traditional predefined sparse transform bases cannot meet dynamic change requirements, Wang et al. proposed a learning-based joint compressed sensing scheme [26], which can learn sparse transform bases from compressed sensing measurement results and utilize spatiotemporal relationships to explore correlations among multiple sources. Although this work has improvements in dynamic adaptability, it is still limited to terrestrial wireless sensor networks and does not consider the special constraints of space–air–ground networks.

### 2.3. Differential Privacy

Differential privacy, as the gold standard for privacy protection, has increasingly mature applications in federated learning. From a theoretical development perspective, a 2024 survey highlighted current challenges faced by differential privacy in practical deployment, including data adaptability, multi-level privacy guarantees, and defense capabilities against other attack types [27]. In specific applications, Guo et al. proposed a differential privacy federated learning framework based on fast Fourier transform [28]. This work reduces the impact of limited computational resources and large numbers of users on training effectiveness by computing privacy budgets through FFT, and uses privacy loss distribution and privacy curves for analysis, reducing the number of manually set hyperparameters.

Considering the reality that different users have different privacy requirements, researchers have proposed hybrid differential privacy mechanisms. Zhang et al. pointed out that using the same privacy protection scheme for all users is inappropriate [29], proposing a hybrid differential privacy federated learning algorithm that provides differentiated privacy protection based on users’ different trust levels toward servers. Liu et al. proposed the AWDP-FL framework, handling model gradient parameters from the neural network layer perspective [30]. In healthcare and other domains with extremely high privacy requirements, differential privacy federated learning applications are more in-depth. Patel et al. combined federated learning with differential privacy for breast cancer detection [31], minimizing performance loss while ensuring strong privacy protection.

### 2.4. Dynamic Routing and Resource Optimization in Space–Air–Ground Networks

The spatiotemporal dynamics of space–air–ground networks pose enormous challenges to traditional network optimization methods. Yang et al. proposed a satellite-assisted task offloading and resource allocation scheme for 6G wide-area edge intelligence [32], considering the dynamics of satellite motion and designing task offloading strategies that perceive dynamic changes. However, this work primarily focuses on computational offloading with limited adaptation to federated learning scenarios. Zhang et al. designed a satellite edge intelligence optimization framework based on deep reinforcement learning [33], utilizing attention mechanisms and multi-agent reinforcement learning to achieve leader federated learning optimization for distributed satellite edge intelligence. Although this work has innovations in system optimization, it lacks comprehensive consideration of privacy protection and communication compression. Considering the trade-off requirements among multiple optimization objectives such as energy consumption, latency, and accuracy in space–air–ground networks, Zhou et al. proposed a multi-objective optimization method based on decomposition and meta-deep reinforcement learning [34]. This method optimizes asynchronous federated learning in 6G satellite systems but does not deeply consider the joint design of compressed sensing and differential privacy.

Through systematic review of related work, we identify four main limitations of existing research. First, compressed sensing methods rely on predefined or static compression matrices that cannot adapt to orbital dynamics; for instance, Jeon et al. [24] achieved strong results in MIMO systems but their matrix design does not exploit satellite orbital periodicity. Second, differential privacy mechanisms adopt fixed budget allocation strategies that fail to leverage orbital predictability for temporal optimization, resulting in budget under-utilization during favorable channel conditions and potential over-consumption during unfavorable ones, as observed even in adaptive works such as AWDP-FL [30]. Third, most approaches model satellite networks as generic mobile ad hoc networks, neglecting the deterministic periodicity of orbital mechanics that is explicitly available through models such as SGP4 [22]. Fourth, multi-objective optimization attempts largely rely on heuristic methods without rigorous theoretical guarantees [34], and none of the existing works provide adaptive mechanisms that jointly exploit orbital spatiotemporal characteristics for simultaneous optimization of compression, privacy, and routing.

To provide a more systematic overview of the above limitations, Table 1 summarizes the key characteristics of representative existing methods compared to the proposed ACDP-FL framework across four critical dimensions: compression strategy, differential privacy mechanism, orbital awareness, and theoretical guarantee.

Regarding reinforcement learning-based routing in space–air–ground networks, existing works have explored this direction from different perspectives. Lozano-Cuadra et al. [23] proposed a continual deep reinforcement learning scheme for decentralized satellite routing, leveraging the predictability of orbital dynamics for policy transfer across constellation reconfigurations. However, their work focuses exclusively on routing optimization and does not integrate privacy protection or communication compression within the federated learning training loop. Zhang et al. [33] adopted multi-agent deep reinforcement learning with attention mechanisms to achieve leader-based federated learning optimization for distributed satellite edge intelligence, representing a more comprehensive integration of RL and FL in SAGIN. Nevertheless, this framework lacks rigorous consideration of differential privacy and treats communication compression as an independent engineering concern rather than a jointly optimized design variable. Zhou et al. [34] further extended the multi-objective optimization dimension through decomposition and meta-deep reinforcement learning, addressing asynchronous federated learning in 6G satellite systems; however, the joint design of compressed sensing and differential privacy remains absent. These gaps collectively motivate the orbital-aware, jointly optimized routing and privacy-compression design proposed in this work.

Beyond these individual limitations, a more fundamental gap exists at the intersection of orbital mechanics and algorithm design. Satellite orbital motion follows deterministic Keplerian dynamics with high temporal predictability: LEO satellites complete an orbit in approximately 90–120 min, and the resulting periodic variation in gradient sparsity patterns, channel quality, and privacy risk exposure are all functions of orbital phase $\phi (t)=2\pi \left(\frac{t}{T_{\mathrm{orbit}}}\;\mathrm{mod}\;1\right)$. Existing compressed sensing methods in federated learning [24,25,26] are designed without reference to this orbital phase structure, meaning their measurement matrices remain static across orbital positions that impose fundamentally different compression requirements. Similarly, differential privacy mechanisms [27,28,29,30] allocate privacy budgets uniformly across rounds, ignoring the fact that privacy risk exposure is systematically higher when satellites pass over sensitive geographic regions and systematically lower during other orbital segments. This paper explicitly bridges this gap by embedding Keplerian orbital state information into both the compressed sensing matrix design (Section 3.2) and the differential privacy budget allocation strategy (Section 3.3), enabling a coherent, orbital-aware federated learning framework that neither prior CS works nor prior DP-FL works have achieved.

## 3. Adaptive Compressed Sensing Differential Privacy Federated Learning

This work considers a three-tier space–air–ground integrated network consisting of geostationary earth orbit (GEO) satellites, medium earth orbit (MEO) satellites, low earth orbit (LEO) satellites, high altitude platforms (HAPs), ground earth stations (GESs), and user terminals (UTs), as specifically illustrated in Figure 1. In this framework, GEO satellites serve as the top-tier global aggregation center, responsible for coordinating the entire federated learning process; MEO/LEO satellites and ground stations constitute the middle tier, performing local aggregation and relay functions; HAPs and user terminals act as bottom-tier nodes, conducting model training based on local data.

### 3.1. System Modeling and Problem Formalization

#### 3.1.1. Space–Air–Ground Network Topology Modeling

We model the space–air–ground integrated network as a time-varying graph G(t)=(V(t),E(t)), where *t* represents time, V(t) denotes the node set at time *t*, and E(t) represents the edge set at time *t*. The node set can be further partitioned into three subsets: $V(t)=V_{\mathrm{space}}(t)\cup V_{\mathrm{air}}(t)\cup V_{\mathrm{ground}}(t)$, representing the space node set, air node set, and ground node set, respectively. The space node set $V_{\mathrm{space}}(t)=V_{\mathrm{GEO}}\cup V_{\mathrm{MEO}}(t)\cup V_{\mathrm{LEO}}(t)$ contains satellite nodes at different orbital altitudes, the air node set $V_{\mathrm{air}}(t)$ contains high altitude platforms, and the ground node set $V_{\mathrm{ground}}$ contains ground stations and user terminals.

For any node i∈V(t), we use $p_{i}(t)={\left[x_{i}(t),y_{i}(t),z_{i}(t)\right]}^{T}$ to represent its three-dimensional position vector in the Earth-centered inertial coordinate system, where $x_{i}(t)$, $y_{i}(t)$, and $z_{i}(t)$ represent the three coordinate components of node *i* at time *t*, respectively. The distance between nodes can be calculated as $d_{ij}(t)=||p_{i}(t)-p_{j}(t)|{|}_{2}$, where $||\cdot |{|}_{2}$ denotes the Euclidean norm. A communication link exists between two nodes *i* and *j* if and only if the distance between them satisfies visibility constraints and the signal strength is sufficient to establish a reliable connection, i.e., (i,j)∈E(t) if and only if $d_{ij}(t)\leq d_{\max}$ and ${\mathrm{SNR}}_{ij}(t)\geq {\mathrm{SNR}}_{\min}$, where $d_{\max}$ represents the maximum communication distance, ${\mathrm{SNR}}_{ij}(t)$ represents the signal-to-noise ratio from node *i* to node *j* at time *t*, and ${\mathrm{SNR}}_{\min}$ represents the minimum signal-to-noise ratio threshold required to establish a reliable connection.

The network connectivity is described by the adjacency matrix $A(t)\in {\left\{0,1\right\}}^{\left|V(t)|\times |V(t)\right|}$, where ${\left[A(t)\right]}_{ij}=1$ if and only if (i,j)∈E(t). To capture the hierarchical characteristics of the network, we further define hierarchical adjacency matrices $A^{\left(l\right)}(t)$, where l∈{0,1,2} represents the bottom tier (HAPs and UTs), middle tier (MEO/LEO satellites and GESs), and top tier (GEO satellites), respectively. This hierarchical modeling facilitates the design of hierarchical federated learning aggregation strategies, where local aggregation is performed within each tier, and then aggregation results are passed to the upper tier.

#### 3.1.2. Orbital Dynamics and Communication Window Modeling

The motion of satellite nodes follows Kepler’s orbital mechanics laws, and their positions can be precisely predicted through orbital six elements. For orbital node *i*, we use $\kappa_{i}={\left[a_{i},e_{i},I_{i},\Omega_{i},\omega_{i},M_{i}\right]}^{T}$ to represent its orbital six elements, where $a_{i}$ represents the semi-major axis, $e_{i}$ represents the eccentricity, $I_{i}$ represents the orbital inclination, $\Omega_{i}$ represents the right ascension of ascending node, $\omega_{i}$ represents the argument of periapsis, and $M_{i}$ represents the mean anomaly. Given the orbital elements at initial time $t_{0}$, the position of node *i* at any time *t* can be obtained by solving the Kepler equation system:

(1) $$p_{i}(t)=R_{z}(\Omega_{i})R_{x}(I_{i})R_{z}(\omega_{i})\left[\begin{array}{c} a_{i}(\cos E_{i}(t)-e_{i})\\ a_{i}\sqrt{1-e_{i}^{2}}\sin E_{i}(t)\\ 0 \end{array}\right]$$

where $E_{i}(t)$ is the eccentric anomaly obtained by solving the Kepler equation $M_{i}(t)=E_{i}(t)-e_{i}\sin E_{i}(t)$, $M_{i}(t)=M_{i}(t_{0})+n_{i}(t-t_{0})$ is the mean anomaly, $n_{i}=\sqrt{\mu /a_{i}^{3}}$ is the mean angular velocity, *μ* is the Earth’s gravitational constant, and $R_{x}(\cdot )$ and $R_{z}(\cdot )$ represent rotation matrices around the *z*-axis and *x*-axis, respectively.

Based on the orbital prediction model, communication windows between any two satellite nodes can be calculated. We define a communication window as a time interval $W_{ij}=[t_{\mathrm{start}}^{\left(ij\right)},t_{\mathrm{end}}^{\left(ij\right)}]$ during which nodes *i* and *j* satisfy visibility constraints. For LEO-GEO links, communication window durations are typically short due to the rapid motion of LEO satellites. We define the reachability function $R_{ij}(t)\in \{0,1\}$ to represent whether nodes *i* and *j* are reachable at time *t*, where $R_{ij}(t)=1$ if and only if there exists an unobstructed line-of-sight path and the minimum elevation angle constraint is satisfied.

Considering propagation delays in practical systems, we further define the effective communication time window $W_{ij}^{\mathrm{eff}}=[t_{\mathrm{start}}^{\left(ij\right)}+\tau_{ij},t_{\mathrm{end}}^{\left(ij\right)}-\tau_{ij}]$, where $\tau_{ij}=d_{ij}/c$ is the signal propagation delay and *c* is the speed of light. The duration of the effective communication window is $T_{ij}^{\mathrm{comm}}=\max(0,t_{\mathrm{end}}^{\left(ij\right)}-t_{\mathrm{start}}^{\left(ij\right)}-2\tau_{ij})$, which represents the time actually available for data transmission.

Link capacity modeling considers free space path loss and atmospheric attenuation effects. For inter-satellite links, the received power can be expressed as

(2) $$P_{ij}^{\mathrm{rx}}(t)=P_{i}^{\mathrm{tx}}G_{i}^{\mathrm{tx}}G_{j}^{\mathrm{rx}}{\left(\frac{\lambda }{4\pi d_{ij}(t)}\right)}^{2}L_{\mathrm{atm}}(t)$$

where $P_{i}^{\mathrm{tx}}$ is the transmit power of node *i*, $G_{i}^{\mathrm{tx}}$ and $G_{j}^{\mathrm{rx}}$ are the transmit and receive antenna gains respectively, *λ* is the carrier wavelength, and $L_{\mathrm{atm}}(t)$ is the atmospheric loss factor. The corresponding signal-to-noise ratio is ${\mathrm{SNR}}_{ij}(t)=P_{ij}^{\mathrm{rx}}(t)/(N_{0}B_{ij})$, where $N_{0}$ is the noise power spectral density and $B_{ij}$ is the bandwidth allocated to link (*i*,*j*). The instantaneous data rate of the link can be calculated according to the Shannon formula:

(3) $$C_{ij}(t)=B_{ij}\log_{2}(1+{\mathrm{SNR}}_{ij}(t))$$

#### 3.1.3. Federated Learning Objective Function Construction

In the space–air–ground federated learning framework, our objective is to train a global model $w^{*}\in {\mathbb{R}}^{d}$ to minimize the overall loss function of local data distributed across various nodes. Let the node set N={1,2,…,N} represent all nodes participating in federated learning, where each node *i* has a local dataset $D_{i}={\left\{(x_{i}^{\left(k\right)},y_{i}^{\left(k\right)})\right\}}_{k=1}^{n_{i}}$, where $x_{i}^{\left(k\right)}\in {\mathbb{R}}^{p}$ is the feature vector of the *k*-th sample, $y_{i}^{\left(k\right)}$ is the corresponding label, and $n_{i}=|D_{i}|$ is the number of samples at node *i*. The local loss function of node *i* is defined as

(4) $$F_{i}(w)=\frac{1}{n_{i}}\sum_{k=1}^{n_{i}}f(w;x_{i}^{\left(k\right)},y_{i}^{\left(k\right)})$$

where f(w;x,y) is the loss function for a single sample (*x*, *y*) and model parameters *w*.

The global optimization objective is to minimize the weighted average loss of all nodes:

(5) $$\underset{w}{\min}\sum_{i=1}^{N}p_{i}F_{i}(w)$$

where $p_{i}=n_{i}/\sum_{j=1}^{N}n_{j}$ is the data weight of node *i*, reflecting the proportion of that node’s data volume in the total. This weighting approach ensures that nodes with more data have greater influence in global model training, which is reasonable in space–air–ground networks since different types of nodes (e.g., GEO satellites vs. user terminals) may have different data collection capabilities.

However, in practical space–air–ground federated learning, inter-node communication is subject to strict bandwidth and energy constraints, with significant propagation delays and intermittent connectivity. Therefore, we need to consider multiple factors including communication efficiency, privacy protection, and energy optimization. The modified objective function can be expressed as

(6) $$\underset{w,\theta }{\min}L(w,\theta )$$

where $L(w,\theta )=F(w)+\lambda_{1}C_{\mathrm{comm}}(\theta )+\lambda_{2}C_{\mathrm{energy}}(\theta )+\lambda_{3}C_{\mathrm{privacy}}(\theta )$ represents algorithm control parameters (such as compression ratio, noise level, etc.), $C_{\mathrm{comm}}(\theta )$, $C_{\mathrm{energy}}(\theta )$, and $C_{\mathrm{privacy}}(\theta )$ represent communication cost, energy cost, and privacy cost respectively, and $\lambda_{1}$, $\lambda_{2}$, $\lambda_{3}$ are corresponding weight coefficients used to balance the importance of different objectives.

The communication cost $C_{\mathrm{comm}}(\theta )$ is mainly determined by the transmission volume of model parameters. In standard federated learning, each client needs to upload complete gradient vectors or model parameters, with communication cost O(d), where *d* is the model parameter dimension. By introducing compressed sensing technology, we can reduce the communication cost to O(s), where s≪d is the dimension of the compressed vector. The energy cost $C_{\mathrm{energy}}(\theta )$ includes both computational energy consumption and communication energy consumption, where communication energy consumption typically dominates, especially for satellite nodes. The privacy cost $C_{\mathrm{privacy}}(\theta )$ quantifies the impact of differential privacy mechanisms on model performance, usually proportional to the variance of injected noise.

#### 3.1.4. Multi-Constraint Optimization Problem Formalization

Considering the special constraints of space–air–ground networks, we formalize the federated learning problem as a multi-constraint optimization problem. Let $w^{\left(t\right)}$ represent the global model parameters in the *t*-th round, $g_{i}^{\left(t\right)}$ represent the local gradient of node *i* in the *t*-th round, and ${\tilde{g}}_{i}^{\left(t\right)}$ represent the gradient after compression and noise processing. The optimization problem can be formulated as

(7) $$\begin{array}{l} \min_{{w^{(t)T}}_{t=0},{\theta^{(t)T}}_{t=0}}\sum_{t=0}^{T}\left[F(w^{\left(t\right)})+\lambda_{1}\sum_{i=1}^{N}{\tilde{||g}}_{i}^{\left(t\right)}{\left|\right|}_{0}+\lambda_{2}E_{\mathrm{total}}^{\left(t\right)}+\lambda_{3}P^{\left(t\right)}\right]\\ \mathrm{subject}\;\mathrm{to}:\\ w^{\left(t+1\right)}=w^{\left(t\right)}-\eta^{\left(t\right)}\sum_{i=1}^{N}p_{i}{\tilde{g}}_{i}^{\left(t\right)},\;\forall t\in \{0,1,\ldots ,T-1\}\\ ∥{\tilde{g}}_{i}^{\left(t\right)}-g_{i}^{\left(t\right)}{∥}_{2}\leq \epsilon_{\mathrm{recon}}^{\left(t\right)},\;\forall i\in \{1,2,\ldots ,N\},\forall t\in \{0,1,\ldots ,T-1\}\\ \sum_{i=1}^{N}∥{\tilde{g}}_{i}^{\left(t\right)}{∥}_{0}\cdot B_{\mathrm{bit}}\leq C_{\mathrm{comm}}^{\left(t\right)},\;\forall t\in \{0,1,\ldots ,T-1\}\\ E_{\mathrm{total}}^{\left(t\right)}\leq E_{\mathrm{budget}}^{\left(t\right)},\;\forall t\in \{0,1,\ldots ,T-1\}\\ P^{\left(t\right)}\leq P_{\mathrm{budget}},\;\forall t\in \{0,1,\ldots ,T-1\}\\ R_{ij}^{\left(t\right)}=1\;\mathrm{if}\;(i,j)\in P_{\mathrm{route}}^{\left(t\right)},\;\forall i,j\in \{1,2,\ldots ,N\},i\neq j,\forall t\in \{0,1,\ldots ,T\} \end{array}$$

where the meanings of each constraint are as follows:

The first constraint is the standard update rule of federated learning, where $\eta^{\left(t\right)}$ is the learning rate in the *t*-th round. This constraint ensures that global model parameters are updated according to federated averaging, but using compressed and privacy-processed gradients ${\tilde{g}}_{i}^{\left(t\right)}$ instead of original gradients $g_{i}^{\left(t\right)}$.

The second constraint is the reconstruction error constraint, limiting the error between the gradient after compressed sensing reconstruction and the original gradient to not exceed the preset threshold $\epsilon_{\mathrm{recon}}^{\left(t\right)}$. This constraint ensures that the compression process does not excessively damage the model’s convergence performance.

The third constraint is the communication capacity constraint, where $||{\tilde{g}}_{i}^{\left(t\right)}|{|}_{0}$ represents the sparsity of the compressed gradient (number of non-zero elements), $B_{\mathrm{bit}}$ is the number of bits per non-zero element, and $C_{\mathrm{comm}}^{\left(t\right)}$ is the total communication capacity budget for the *t*-th round. This constraint ensures that the communication requirements of all nodes do not exceed the actual network capacity.

The fourth constraint is the energy budget constraint, where $E_{\mathrm{total}}^{\left(t\right)}=\sum_{i=1}^{N}\left(E_{i,\mathrm{comp}}^{\left(t\right)}+E_{i,\mathrm{comm}}^{\left(t\right)}\right)$ represents the total energy consumption in the *t*-th round, including computational energy consumption $E_{i,\mathrm{comp}}^{\left(t\right)}$ and communication energy consumption $E_{i,\mathrm{comm}}^{\left(t\right)}$ for all nodes, and $E_{\mathrm{budget}}^{\left(t\right)}$ is the energy budget.

The fifth constraint is the privacy budget constraint, where $P^{\left(t\right)}$ represents the privacy loss in the *t*-th round, and $P_{\mathrm{budget}}$ is the total privacy budget. Under the differential privacy framework, privacy loss is cumulative, so it is necessary to ensure that the total privacy loss throughout the entire training process does not exceed the preset threshold.

The sixth constraint is the routing reachability constraint, ensuring that all links in the communication path $P_{\mathrm{route}}^{\left(t\right)}$ selected in the *t*-th round are reachable at the corresponding time.

The total energy consumption $E_{\mathrm{total}}^{\left(t\right)}$ can be further decomposed as

(8) $$E_{\mathrm{total}}^{\left(t\right)}=\sum_{i=1}^{N}\left[\alpha_{i}∥g_{i}^{\left(t\right)}{∥}_{2}^{2}+\beta_{i}∥{\tilde{g}}_{i}^{\left(t\right)}{∥}_{0}+\gamma_{i}P_{i}^{\mathrm{tx}}T_{i,\mathrm{comm}}^{\left(t\right)}\right]$$

where the first term $\alpha_{i}∥g_{i}^{\left(t\right)}{∥}_{2}^{2}$ represents the computational energy consumption of node *i*, proportional to the complexity of gradient calculation; the second term $\beta_{i}∥{\tilde{g}}_{i}^{\left(t\right)}{∥}_{0}$ represents the energy consumption of compression processing, proportional to the number of non-zero elements to be transmitted; the third term $\gamma_{i}P_{i}^{\mathrm{tx}}T_{i,\mathrm{comm}}^{\left(t\right)}$ represents the communication energy consumption, where $P_{i}^{\mathrm{tx}}$ is the transmit power of node *i* and $T_{i,\mathrm{comm}}^{\left(t\right)}$ is the communication time of node *i* in the *t*-th round.

The privacy loss $P^{\left(t\right)}$ under the (*ε*,*δ*)-differential privacy framework can be expressed as

(9) $$P^{\left(t\right)}=\sum_{i=1}^{N}\epsilon_{i}^{\left(t\right)}$$

where $\epsilon_{i}^{\left(t\right)}$ is the privacy parameter of node *i* in the *t*-th round. For the Gaussian mechanism, the relationship between privacy parameters and noise level is $\epsilon_{i}^{\left(t\right)}=\frac{\Delta_{i}^{\left(t\right)}}{\sigma_{i}^{\left(t\right)}}\sqrt{2\log(1.25/\delta )}$, where $\Delta_{i}^{\left(t\right)}$ is the global sensitivity of node *i* in the *t*-th round, $\sigma_{i}^{\left(t\right)}$ is the standard deviation of the added Gaussian noise, and *δ* is the second parameter of differential privacy.

In the space–air–ground network environment, participating nodes (GEO/MEO/LEO satellites, HAPs, ground terminals) collect data from geographically distinct coverage areas, resulting in naturally non-IID data distributions. To quantify the degree of data heterogeneity, we adopt the Dirichlet distribution parameterization $\mathrm{Dir}(\beta_{\mathrm{het}})$ commonly used in federated learning research, where a smaller $\beta_{\mathrm{het}}$ corresponds to more severe heterogeneity. The heterogeneity level of node *i* relative to the global distribution can be measured by the Earth Mover’s Distance (EMD):

(10) $${\mathrm{EMD}}_{i}=\sum_{c=1}^{C}\left|p_{i}^{c}-p_{\mathrm{global}}^{c}\right|\;$$

where *C* is the number of classes, $p_{i}^{c}$ is the proportion of class c in node *i*’s local dataset, and $p_{\mathrm{global}}^{c}$ is the global class proportion. In our simulation, nodes are assigned data using $\mathrm{Dir}(\beta_{\mathrm{het}}=0.5)$, yielding average EMD values of 0.31 for ground terminals, 0.24 for LEO/HAP nodes, and 0.18 for MEO/GEO nodes, reflecting the natural geographic stratification of satellite coverage. This non-IID configuration is reported explicitly in Section 4.1 to ensure reproducibility. The performance gap between local and global objectives under this heterogeneity level satisfies the bounded gradient dissimilarity assumption used in Theorem 1, with dissimilarity parameter $\zeta^{2}$ bounded as

(11) $$\zeta^{2}=\frac{1}{N}\sum_{i=1}^{N}{\left\|\nabla F_{i}(w)-\nabla F(w)\right\|}^{2}\leq G^{2}$$

where *G* is a finite constant estimated empirically as *G* ≈ 0.42 in our experimental setup, confirming the validity of the convergence analysis in Theorem 1 under the observed non-IID conditions.

Solving this multi-constraint optimization problem requires simultaneous consideration of multiple factors including model performance, communication efficiency, energy limitations, privacy protection, and network reachability, making it a complex non-convex optimization problem. The following sections will detail the spatiotemporal adaptive compressed sensing and differential privacy mechanisms, as well as corresponding joint optimization algorithms to effectively solve this problem.

### 3.2. Spatiotemporal Adaptive Compressed Sensing Mechanism

Traditional compressed sensing methods typically employ fixed sparse transform bases and static measurement matrices, which exhibit obvious deficiencies when facing the dynamic characteristics of space–air–ground networks. To fully exploit the periodicity and predictability of satellite orbital motion, we propose a spatiotemporal adaptive compressed sensing mechanism that can dynamically adjust compression strategies based on orbital position, communication windows, and channel conditions, maximizing communication efficiency while ensuring reconstruction accuracy.

#### 3.2.1. Orbital Periodicity-Driven Sparse Sensing Matrix Design

The deterministic nature of satellite orbital motion provides important prior information for adaptive design of compressed sensing matrices. We construct time-varying sparse sensing matrices based on orbital periodicity characteristics to capture sparsity patterns of gradient vectors at different orbital positions. Let the orbital phase of satellite node *i* at time *t* be $\phi_{i}(t)=2\pi \left(\frac{t}{T_{i}}-\left\lfloor \frac{t}{T_{i}}\right\rfloor \right)$, where *T_i_* is the orbital period of node *i*. Based on the periodic variation in orbital phase, we divide the orbit into *K* equally spaced phase intervals, with each interval corresponding to a specific sparsity pattern. For the *k*-th phase interval $\Phi_{k}=\left[\frac{2\pi (k-1)}{K},\frac{2\pi k}{K}\right)$, we define the corresponding basic sparsity pattern $S_{k}\in {\left\{0,1\right\}}^{d}$, where ${\left[S_{k}\right]}_{j}\in \{0,1\}$ indicates the importance of the *j*-th parameter, and *d* is the model parameter dimension. This pattern is obtained through statistical analysis of historical orbital data, specifically calculated as

(12) $$S_{k}=I\left(\frac{1}{\left|H_{k}\right|}\sum_{t\in H_{k}}|g_{i}(t)|>\tau_{\mathrm{sparse}}\right)$$

where $H_{k}$ represents the set of historical time instances in phase interval $\Phi_{k}$, $g_{i}(t)$ represents the gradient vector of node *i* at time *t*, and $\tau_{\mathrm{sparse}}$ is the sparsity threshold.

To achieve smooth transitions between adjacent phase intervals and avoid performance degradation due to abrupt changes, we introduce a trigonometric function-based interpolation weighting mechanism. When the orbital phase of node *i* at time *t* is $\phi_{i}(t)$, the adaptive sparsity pattern is calculated as

(13) $$S_{i}(t)=\sum_{k=1}^{K}w_{k}(\phi_{i}(t))S_{k}$$

where the weight function $w_{k}(\phi )$ is defined as

(14) $$w_{k}(\phi )=\max\left(0,1-\frac{K}{2\pi }\min\left(\left|\phi -\phi_{k}|,2\pi -|\phi -\phi_{k}\right|\right)\right)$$

Here $\phi_{k}=\frac{2\pi (k-0.5)}{K}$ is the center phase of the *k*-th phase interval. This weight function ensures that only 2–3 adjacent basic patterns participate in interpolation at each moment, thereby maintaining local consistency of sparsity.

Based on the adaptive sparsity pattern, we construct the time-varying compressed sensing measurement matrix $\Phi_{i}(t)\in {\mathbb{R}}^{m_{i}(t)\times d}$. This matrix consists of two parts: a structured part and a randomized part. The structured part $\Phi_{i}^{\mathrm{struct}}(t)$ performs dense sampling specifically for identified important parameter positions, expressed as

(15) $$\Phi_{i}^{\mathrm{struct}}(t)=P_{S_{i}(t)}\Psi_{\mathrm{DCT}}^{T}$$

where $P_{S_{i}(t)}\in {\mathbb{R}}^{s_{i}(t)\times d}$ is the selection matrix based on sparsity pattern *S_i_*(*t*), $s_{i}(t)=||S_{i}(t)|{|}_{0}$ is the number of identified important parameters, and $\Psi_{\mathrm{DCT}}$ is the discrete cosine transform matrix used to capture frequency-domain sparsity of gradients. The randomized part $\Phi_{i}^{\mathrm{rand}}(t)\in {\mathbb{R}}^{r_{i}(t)\times d}$ uses a Gaussian random matrix with elements independently and identically distributed as N(0,1/d), designed to capture unexpected sparsity patterns, and *s_i_*(*t*) represents the number of important parameters (sparsity) for node *i* at time *t*. The final measurement matrix is expressed as

(16) $$\Phi_{i}(t)=\left[\begin{array}{c} \begin{array}{l} \alpha_{i}(t)\Phi_{i}^{\mathrm{struct}}(t)\;\\ \beta_{i}(t)\Phi_{i}^{\mathrm{rand}}(t) \end{array} \end{array}\right]$$

where $\alpha_{i}(t)$ and $\beta_{i}(t)$ are normalization coefficients satisfying $\alpha_{i}{\left(t\right)}^{2}+\beta_{i}{\left(t\right)}^{2}=1$.

In practical deployment, the orbital phase $\phi_{i}(t)$ used to drive the sparsity pattern selection in Equation (13) is computed directly from Two-Line Element (TLE) data. Given TLE parameters for satellite node *i*, the orbital period $T_{i}$ and mean anomaly $M_{i}(t)$ are propagated forward using the SGP4/SDP4 model, yielding a real-time orbital phase estimate. The phase interval index *k* is then determined as

(17) $$k=\left\lfloor \frac{\phi_{i}(t)}{2\pi /K}\right\rfloor +1$$

where *K* is the total number of phase intervals. The rationale for this discretization is that gradient sparsity patterns in satellite-borne tasks, such as remote sensing image classification, exhibit statistically stable structure within each phase segment due to the periodic variation in ground coverage and data distribution. Specifically, when a LEO satellite transits over high-density data regions (e.g., urban or coastal areas), the gradient vectors tend to concentrate energy in spatial-frequency components corresponding to high-frequency textures, yielding a different sparsity signature than when the satellite is over ocean or polar regions. This orbital phase-dependent sparsity structure is captured by the basic sparsity pattern $S_{k}$ defined in Equation (12), which is estimated as offline from historical gradient statistics and updated periodically as new orbital data becomes available. The structured measurement component $\Phi_{i}^{\mathrm{struct}}(t)$ in Equation (15) therefore encodes orbital domain knowledge directly into the CS matrix, providing a principled link between Keplerian orbital mechanics and compressed sensing design that distinguishes the proposed approach from static-CS methods [24,25].

#### 3.2.2. Dynamic Compression Rate Adjustment and Error Bounds

The choice of compression rate directly affects the trade-off between communication overhead and reconstruction accuracy. In space–air–ground networks, communication conditions change dynamically with orbital motion, necessitating adaptive adjustment of compression rates to suit current network states. We design a dynamic compression rate adjustment strategy based on communication window prediction and channel quality assessment.

Let the target compression rate for node *i* in the *t*-th training round be $\rho_{i}(t)\in (0,1]$, then the number of measurements after compression is $m_{i}(t)=\left\lceil \rho_{i}(t)\cdot d\right\rceil$, where *d* is the total number of model parameters. The determination of compression rate requires comprehensive consideration of three factors: communication window constraints, channel quality, and reconstruction accuracy requirements. This work employs a multi-objective optimization method to determine the optimal compression rate. The utility function is defined as

(18) $$U_{i}(t,\rho )=\omega_{1}(1-\rho )+\omega_{2}{\bar{Q}}_{i}(t)-\omega_{3}I(\rho <\rho_{\min,i}^{\left(t\right)})$$

where $\omega_{1},\omega_{2},\omega_{3}$ are weight coefficients, the first term encourages higher compression rates to reduce communication overhead, the second term considers the impact of channel quality, the third term is a penalty for reconstruction accuracy constraints, and ${\bar{Q}}_{i}(t)=\min_{j\in A_{i}(t)}Q_{ij}(t)$ is the worst link quality of node *i*. The optimal compression rate is obtained by solving the following optimization problem:

(19) $$\rho_{i}^{*}(t)=\arg\underset{\rho \in [\rho_{\min,i}^{\left(t\right)},\rho_{\max,i}^{\left(t\right)}]}{\max}U_{i}(t,\rho )$$

where $\rho_{\min,i}^{\left(t\right)}$ represents the lower bound determined by reconstruction accuracy constraints, and $\rho_{\max,i}^{\left(t\right)}$ represents the upper bound determined by communication capacity constraints.

To guarantee theoretical convergence of the federated learning algorithm, we need to establish theoretical bounds for compressed sensing reconstruction errors. Under the spatiotemporal adaptive compressed sensing framework, reconstruction errors are affected by multiple factors including sparsity assumptions, measurement noise, and algorithm accuracy.

Under the condition that the adaptive measurement matrix satisfies the Restricted Isometry Property (RIP), the reconstruction error upper bound using the *ℓ*_1_ minimization reconstruction algorithm is

(20) $$∥{\hat{g}}_{i}-g_{i}{∥}_{2}\leq C_{1}\cdot \frac{∥g_{i}-g_{i}^{\left(s\right)}{∥}_{1}}{\sqrt{s}}+C_{2}\cdot \frac{∥n_{i}{∥}_{2}}{\sqrt{m_{i}(t)}}$$

where ${\hat{g}}_{i}$ is the reconstructed gradient vector of node *i*, *g_i_* is the true gradient vector of node *i*, $g_{i}^{\left(s\right)}$ is the best *s*-term sparse approximation of *g_i_*, *n_i_* is the measurement noise vector, *C*_1_ and *C*_2_ are constants, and $m_{i}(t)$ is the measurement dimension of node *i* at time *t*.

Under the spatiotemporal adaptive framework, considering the influence of historical sparsity pattern prediction error $\epsilon_{pred}$, the reconstruction error bound is modified to

(21) $$∥{\hat{g}}_{i}-g_{i}{∥}_{2}\leq C_{1}\cdot \frac{\sigma_{1}(g_{i})}{\sqrt{s}}+C_{2}\cdot \frac{\sigma_{noise}}{\sqrt{m_{i}(t)}}+C_{3}\cdot \epsilon_{pred}$$

where $\sigma_{1}(g_{i})$ represents the sparse approximation error of *g_i_*, $\epsilon_{pred}$ is the historical sparsity pattern prediction error, and *C*_3_ is a coefficient reflecting the degree of influence of prediction errors. This result indicates that orbital periodicity-driven sparsity pattern design can improve reconstruction accuracy by reducing $\epsilon_{pred}$.

Channel conditions in space–air–ground networks vary dynamically with time and node positions, directly affecting the performance of compressed sensing systems. We design a time-varying channel condition-aware optimization strategy that maintains system performance stability by real-time monitoring of channel parameters and corresponding adjustment of compressed sensing parameters.

Based on the deterministic nature of orbital dynamics, we establish the channel quality metric *Q_i_ⱼ*(t) as follows:

(22) $$Q_{ij}(t)=w_{1}\cdot \frac{SNR_{ij}(t)}{SNR_{ref}}+w_{2}\cdot \left(1-\frac{d_{ij}(t)}{d_{max}}\right)+w_{3}\cdot \left(1-\rho_{ij}(t)\right)$$

where *SNR*_i_ⱼ(*t*) represents the signal-to-noise ratio of link (*i*,*j*) at current time *t*, *d_i_ⱼ*(*t*) represents the Euclidean distance between nodes *i* and *j* at time *t*, $\rho_{ij}(t)$ is the load rate of link (*i*,*j*) at time t; *w*_1_, *w*_2_, *w*_3_ are weighting coefficients satisfying *w*_1_ + *w*_2_ + *w*_3_ = 1, representing the importance of signal-to-noise ratio, distance, and load rate respectively, $SNR_{ref}$ is the reference signal-to-noise ratio, and *d_max_* is the maximum communication distance.

Based on the channel quality metric, the adaptive adjustment formula for compression rate is

(23) $$\rho_{i}(t)=\rho_{base}\cdot \left(1+\alpha \cdot \tanh\left(\beta \cdot \frac{Q_{i}(t)-Q_{ref}}{\sigma_{Q}}\right)\right)$$

where $\rho_{base}$ is the base compression rate, *α* is the parameter controlling compression rate adjustment amplitude, controlling the maximum range of compression rate changes, *β* is the sensitivity parameter for quality changes, controlling the sensitivity of quality changes to compression rate adjustment, $Q_{ref}$ is the reference channel quality, typically set as the historical average quality value, and $\sigma_{Q}$ is the standard deviation of channel quality metrics, used for normalizing quality changes.

To handle sudden channel deterioration, the system activates emergency mechanisms through anomaly detection:

(24) $$\mathrm{Anomaly}\;\mathrm{Detection}=I\left({∥g_{i}^{recon}∥}_{2}>\lambda \cdot \mu_{hist}\;\mathrm{or}\;CRC({\tilde{y}}_{i})\neq \mathrm{True}\right)$$

where $I\left(•\right)$ is the indicator function returning 1 when the condition is true and 0 otherwise, $g_{i}^{recon}$ is the reconstructed gradient vector, $\mu_{hist}$ is the historical mean of node *i*’s gradient norm, calculated based on a sliding window.

When transmission errors are detected, the system activates degraded transmission mode, transmitting only the most important components of gradients:

(25) $$g_{i}^{degraded}=TopK(g_{i},k_{emergency})$$

where $g_{i}^{degraded}$ is the simplified gradient vector in degraded mode, TopK(·,k) represents retaining the top *k* emergency elements with largest absolute values, and $k_{emergency}$ is the number of parameters retained in emergency mode.

Through the above time-varying channel condition-aware mechanism, the system can maintain efficient gradient compression and reliable reconstruction performance in the dynamic space–air–ground network environment.

### 3.3. Orbital-Aware Differential Privacy Mechanism

Traditional differential privacy mechanisms typically employ fixed privacy budget allocation strategies, which exhibit obvious deficiencies in the dynamic space–air–ground network environment. Satellite node orbital motion possesses high predictability, and communication conditions, data importance, and privacy risks under different orbital positions exhibit periodic variation patterns. Based on this observation, we propose an orbital-aware differential privacy mechanism that can dynamically adjust privacy protection strategies according to orbital states, link quality, and path characteristics, maximizing model performance while ensuring privacy security.

#### 3.3.1. Temporal Privacy Budget Allocation Based on Orbital Predictability

The deterministic nature of satellite orbits provides important opportunities for intelligent allocation of privacy budgets. We design a temporal-aware privacy budget allocation strategy based on orbital periodicity, fully exploiting orbital prediction information to optimize the usage efficiency of privacy resources.

Orbital Periodicity Modeling: Let the orbital period of satellite node *i* be *T_i_*. We divide one complete orbital period into *M* equal-duration time segments, with each time segment $\Delta t=T_{i}/M$ corresponding to a specific orbital state. We define the orbital time index $\tau_{i}(t)=\left\lfloor \frac{t\;\mathrm{mod}\;T_{i}}{\Delta t}\right\rfloor$, representing the orbital time segment corresponding to time *t*. Based on historical data analysis, we define the basic privacy requirement coefficient $\alpha_{\tau }\in [0,1]$ for each orbital time segment τ, which reflects the privacy risk level at different orbital positions. The specific calculation is as follows:

(26) $$\alpha_{\tau }=\frac{1}{\left|H_{\tau }\right|}\sum_{t\in H_{\tau }}\left[w_{\mathrm{exposure}}E_{\mathrm{geo}}(t)+w_{\mathrm{sensitivity}}S_{\mathrm{data}}(t)+w_{\mathrm{attack}}R_{\mathrm{attack}}(t)\right]$$

where $H_{\tau }$ represents the set of historical time instances in orbital time segment τ, $E_{\mathrm{geo}}(t)\in [0,1]$ is the geographical exposure (reflecting the degree to which satellites cover sensitive areas), $S_{\mathrm{data}}(t)\in [0,1]$ is data sensitivity (based on mission type and data content), $R_{\mathrm{attack}}(t)\in [0,1]$ is attack risk (based on threat intelligence and historical attack records), and $w_{\mathrm{exposure}}$, $w_{\mathrm{sensitivity}}$, $w_{\mathrm{attack}}$ are corresponding weight coefficients satisfying $w_{\mathrm{exposure}}+w_{\mathrm{sensitivity}}+w_{\mathrm{attack}}=1$.

Dynamic Budget Allocation Strategy: Based on orbital periodicity modeling, we design a privacy budget allocation strategy. Let the total privacy budget of node *i* be $B_{i}$, which needs to be allocated across *T* rounds during the entire training process. The traditional uniform allocation strategy assigns $\epsilon_{i}^{\mathrm{uniform}}=B_{i}/T$ privacy budget per round, while our orbital-aware allocation strategy considers the time-varying characteristics of orbital states.

First, we predict the orbital state sequence ${\left\{\tau_{i}(t+k)\right\}}_{k=1}^{W}$ for node *i* within the next *W* communication rounds, and calculate the corresponding privacy requirement weight sequence ${\left\{\alpha_{\tau_{i}(t+k)}\right\}}_{k=1}^{W}$. Then, based on the current remaining budget $B_{i}^{\mathrm{remain}}(t)$ and remaining rounds $T^{\mathrm{remain}}(t)=T-t$, we calculate the privacy budget allocation for the *t*-th round as follows:

(27) $$\epsilon_{i}^{\left(t\right)}=\frac{B_{i}^{\mathrm{remain}}(t)}{Z_{i}(t)}\cdot \alpha_{\tau_{i}(t)}\cdot \beta_{\mathrm{urgency}}(t)$$

where the normalization factor $Z_{i}(t)$ is defined as

(28) $$Z_{i}(t)=\sum_{k=0}^{\min(W-1,T^{\mathrm{remain}}(t)-1)}\alpha_{\tau_{i}(t+k)}\cdot \gamma^{k}$$

Here *γ* ∈ (0, 1] is the temporal decay factor used to reduce the weight of long-term predictions. The urgency adjustment factor $\beta_{\mathrm{urgency}}(t)$ is calculated as

(29) $$\beta_{\mathrm{urgency}}(t)=\max\left(1,\sqrt{\frac{T^{\mathrm{remain}}(t)}{B_{i}^{\mathrm{remain}}(t)/{\bar{\epsilon }}_{i}}}\right)$$

where ${\bar{\epsilon }}_{i}=B_{i}/T$ is the average privacy budget. This factor ensures increased allocation when privacy budget consumption is too fast and decreased allocation when consumption is too slow.

Budget Smoothing Mechanism: To avoid dramatic fluctuations in budget allocation, we introduce an exponential moving average smoothing mechanism as follows:

(30) $$\epsilon_{i}^{\left(t\right)}=(1-\lambda_{\mathrm{smooth}})\epsilon_{i}^{\left(t-1\right)}+\lambda_{\mathrm{smooth}}{\tilde{\epsilon }}_{i}^{\left(t\right)}$$

where ${\tilde{\epsilon }}_{i}^{\left(t\right)}$ is the original allocation value calculated by the above formula, and $\lambda_{\mathrm{smooth}}\in (0,1)$ is the smoothing coefficient. Meanwhile, we set safety boundary constraints as follows:

(31) $$\epsilon_{\min}\leq \epsilon_{i}^{\left(t\right)}\leq \min\left(\epsilon_{\max},\frac{B_{i}^{\mathrm{remain}}(t)}{T^{\mathrm{remain}}(t)}\right)$$

The above settings ensure that privacy budget usage is neither too conservative nor too aggressive.

To formally justify the orbital-aware privacy budget allocation, we now establish that the proposed mechanism satisfies (*ε*, *δ*)-differential privacy under the Gaussian mechanism framework. Let M denote the overall privatization mechanism applied to the gradient of node *i* in round *t*. The mechanism first compresses the gradient via $y_{i}^{\left(t\right)}=\Phi_{i}^{\left(t\right)}g_{i}^{\left(t\right)}$, then injects Gaussian noise with adaptive standard deviation $\sigma_{i}^{\left(t\right)}$ as defined in Equation (47). By the properties of the Gaussian mechanism [27], the per-round privacy cost satisfies:

(32) $$\varepsilon_{i}^{\left(t\right)}=\frac{\Delta_{i}^{\mathrm{comp}(t)}}{\sigma_{i}^{\left(t\right)}}\sqrt{2\ln\left(\frac{1.25}{\delta }\right)}\;$$

where $\Delta_{i}^{\mathrm{comp}(t)}$ is the global sensitivity in the compressed domain defined in Equation (46). Substituting the adaptive noise standard deviation from Equation (47) into (32) confirms that the allocated budget $\varepsilon_{i}^{\left(t\right)}$ from Equation (27) is exactly consumed, i.e., the mechanism is ($\varepsilon_{i}^{\left(t\right)}$, *δ*)-differentially private per round. Over T training rounds, composition under the advanced composition theorem [27] gives a total privacy cost for node *i*:

(33) $$\varepsilon_{i}^{\mathrm{total}}=\sum_{t=0}^{T-1}\varepsilon_{i}^{\left(t\right)}\leq \varepsilon_{i}$$

where the inequality holds by construction of the normalization factor $Z_{i}(t)$ in Equation (28), which ensures $\sum_{t}\varepsilon_{i}^{\left(t\right)}=\varepsilon_{i}$. This establishes that the orbital-aware budget allocation does not weaken the total privacy guarantee relative to uniform allocation; it only redistributes the budget across rounds according to orbital risk levels $\alpha_{\tau }$ defined in Equation (26). The key insight is that rounds with high privacy risk (high $\alpha_{\tau }$, e.g., satellite over sensitive regions) receive larger budget allocations, enabling stronger noise injection precisely when eavesdropping risk is elevated, while rounds with low risk receive smaller allocations to preserve model accuracy during favorable orbital phases.

Regarding multi-hop privacy accumulation, the model in Equation (38) is a conservative upper bound consistent with the sequential composition theorem of differential privacy [28]. For a path $\Pi =\{v_{0},v_{1},\ldots ,v_{L}\}$ of length *L*, each intermediate node $v_{i}$ applies an independent ($\varepsilon_{v_{i}}$, *δ*)-DP mechanism. By sequential composition, the total privacy loss satisfies

(34) $$\varepsilon_{\Pi }^{\mathrm{total}}\leq \sum_{i=0}^{L}\varepsilon_{v_{i}}$$

The correlation correction factor $\gamma \cdot \mathrm{corr}(v_{i},v_{j})$ in Equation (38) is a conservative reduction term that accounts for the fact that correlated nodes (close in orbit or geography) provide partially redundant privacy protection, yielding a tighter bound than naive sequential composition. While this correction is heuristically motivated, it is conservative in the sense that γ·corr(·)∈[0,1], ensuring the bound in Equation (38) never exceeds the standard sequential composition result in (38). A formal tighter bound exploiting inter-node correlation remains an open theoretical question and is identified as future work.

#### 3.3.2. Adaptive Noise Injection and Privacy Optimization

Channel conditions in space–air–ground networks directly affect the effectiveness and necessity of differential privacy noise. This work designs a link quality-aware adaptive noise injection mechanism and establishes a privacy leakage accumulation model under multi-hop paths, achieving intelligent balance between privacy protection and model performance.

We establish a comprehensive link quality metric to guide noise injection strategies as follows:

(35) $$Q_{ij}^{comp}(t)=w_{1}^{\prime }\cdot \frac{SNR_{ij}(t)}{SNR_{ref}}+w_{2}^{\prime }\cdot \left(1-\frac{BER_{ij}(t)}{BER_{max}}\right)+w_{3}^{\prime }\cdot \left(1-\frac{\tau_{ij}(t)}{\tau_{max}}\right)$$

where $Q_{ij}^{comp}(t)$ represents the comprehensive quality metric of link (*i*,*j*) at time *t* with value range [0, 1], $w_{1}^{\prime }$, $w_{2}^{\prime }$, $w_{3}^{\prime }$ are weight coefficients satisfying $w_{1}^{\prime }+w_{2}^{\prime }+w_{3}^{\prime }=1$, representing the importance of signal-to-noise ratio, bit error rate, and delay respectively, BER*_i_ⱼ*(*t*) represents the bit error rate of link (*i*,*j*) with value range [0, 1], BER*_max_* is the acceptable maximum bit error rate, and $\tau_{ij}(t)$ represents the propagation delay of link (*i*,*j*) in milliseconds.

Based on link quality assessment, this work designs a quality-aware noise scaling mechanism. For a given privacy budget $\epsilon_{i}(t)$, the adaptive noise standard deviation is

(36) $$\sigma_{i}^{adaptive}(t)=\frac{GS_{i}^{comp}(t)}{\epsilon_{i}(t)}\cdot \sqrt{2\cdot \ln\left(\frac{1.25}{\delta }\right)}\cdot \zeta_{i}(t)$$

where $GS_{i}^{comp}(t)$ represents the global sensitivity in the compressed domain, $\epsilon_{i}(t)$ is the privacy budget allocated to node *i* in the *t*-th round, and $\zeta_{i}(t)$ is the adaptive function defined as follows:

(37) $$\zeta_{i}(t)=\left(1-\alpha_{q}\cdot Q_{i}^{avg}(t)\right)\cdot \left(1+\alpha_{c}\cdot \max\left(0,\frac{∥g_{i}^{recon}-g_{i}{∥}_{2}}{∥g_{i}{∥}_{2}}-\sigma_{ref}\right)\right)$$

where $\alpha_{q}$ represents the quality adjustment parameter controlling the degree of link quality influence on noise, $Q_{i}^{avg}(t)$ is the average link quality of node *i* at time *t*, and $\alpha_{c}$ is the compression error adjustment parameter.

In multi-hop transmission environments, we establish a privacy loss accumulation model. Let the path from source node *s* to target node *d* be $P=\{v_{0},v_{1},\ldots ,v_{L}\}$, where the total privacy loss on the path is

(38) $$\epsilon_{total}^{P}=\sum_{i=0}^{L-1}\epsilon_{v_{i}}\cdot \left(1-\gamma \cdot \min_{j\neq i}\mathrm{corr}(\epsilon_{v_{i}},\epsilon_{v_{j}})\right)$$

where $\epsilon_{total}^{P}$ is the total privacy loss on path P, *L* is the path length (number of hops), $v_{i}$ represents the *i*-th node on the path, $\epsilon_{v_{i}}$ is the privacy parameter at node $v_{i}$, γ represents the correlation correction factor, and the privacy correlation coefficient between adjacent hops is calculated based on geographical location and temporal correlation.

We establish a trade-off optimization problem between privacy protection strength and model accuracy. Under given privacy and communication constraints, the multi-objective optimization problem is

(39) $$\min_{\epsilon ,\rho }\left\{f_{1}(\theta )=E[L(\theta )],\;f_{2}(\epsilon )=\sum_{i=1}^{N}\epsilon_{i}\cdot R_{i}(t)\right\}$$

where $\epsilon ={\left[\epsilon_{1},\ldots ,\epsilon_{N}\right]}^{T}$ represents the privacy budget vector, *ρ* is the compression rate vector, $f_{1}(\theta )$ is the model performance objective function representing the expected value of global loss, $f_{2}(\epsilon )$ is the privacy cost function considering orbital position privacy risks, L(θ) represents the loss function based on global model parameters *θ*, and *R_i_*(*t*) is the orbital privacy risk coefficient of node *i* at time *t*.

We achieve solution selection on the Pareto frontier through dynamic weight adjustment:

(40) $$\epsilon^{*(t)},\;\rho^{*(t)}=\arg\min_{\epsilon ,\;\rho }\left[w_{p}(t)\cdot f_{1}(\theta )+w_{a}(t)\cdot f_{2}(\epsilon )\right]$$

where $\epsilon^{*(t)}$ and $\rho^{*(t)}$ represent the optimal privacy budget and compression rate at time *t* respectively, and $w_{p}(t)$ and $w_{a}(t)$ are time-varying weight coefficients satisfying $w_{p}(t)+w_{a}(t)=1$.

The dynamic weight adjustment strategy is based on training progress and orbital periodicity:

(41) $$w_{p}(t)=w_{p}^{base}+A_{p}\cdot \sin\left(\frac{2\pi t}{T_{orbit}}\right)+B_{p}\cdot \frac{t}{T_{total}}$$

where $w_{p}^{base}$ represents the base accuracy weight, $A_{p}$ is the amplitude coefficient for periodic adjustment, $T_{orbit}$ represents the average orbital period used to capture orbital periodic variations, $B_{p}$ is the linear growth coefficient making the accuracy weight gradually increase in the later stages of training, and $T_{total}$ is the total number of training rounds.

Through the above adaptive noise injection and privacy optimization mechanisms, the system achieves intelligent balance between privacy protection and model performance.

### 3.4. Compression–Privacy Joint Optimization

#### 3.4.1. Differential Privacy Noise Injection Strategy in Compressed Domain

Traditional differential privacy mechanisms typically add noise in the original data space. However, under the compressed sensing framework, this approach suffers from two main problems: first, the high dimensionality of the original gradient space requires substantial noise to ensure privacy, which significantly degrades model performance; second, the compression process may amplify or attenuate noise effects, undermining the theoretical guarantees of differential privacy. To address these issues, this work designs a differential privacy noise injection strategy specifically tailored for the compressed domain.

Let the original gradient of node *i* in the *t*-th round be $g_{i}^{\left(t\right)}\in {\mathbb{R}}^{d}$, and the measurement vector after adaptive compressed sensing be $y_{i}^{\left(t\right)}=\Phi_{i}^{\left(t\right)}g_{i}^{\left(t\right)}\in {\mathbb{R}}^{m_{i}^{\left(t\right)}}$, where $\Phi_{i}^{\left(t\right)}\in {\mathbb{R}}^{m_{i}^{\left(t\right)}\times d}$ is the time-varying measurement matrix and $m_{i}^{\left(t\right)}=\left\lceil \rho_{i}^{\left(t\right)}\cdot d\right\rceil$ is the number of measurements after compression. The process of adding differential privacy noise in the compressed domain can be expressed as follows:

(42) $${\tilde{y}}_{i}^{\left(t\right)}=y_{i}^{\left(t\right)}+\eta_{i}^{\left(t\right)}$$

where $\eta_{i}^{\left(t\right)}\in {\mathbb{R}}^{m_{i}^{\left(t\right)}}$ is the injected privacy noise vector. To guarantee the theoretical properties of differential privacy, each component of the noise vector must be independently and identically sampled from a Gaussian distribution: ${\left[\eta_{i}^{\left(t\right)}\right]}_{j}\sim N(0,{\left(\sigma_{i}^{\left(t\right)}\right)}^{2}),\;j=1,2,\ldots ,m_{i}^{\left(t\right)}$.

The key challenge of differential privacy in the compressed domain lies in determining the appropriate noise standard deviation $\sigma_{i}^{\left(t\right)}$. According to differential privacy theory, the noise standard deviation is directly related to global sensitivity. Under the compressed sensing framework, the global sensitivity after compression is defined as follows:

(43) $$\Delta_{i}^{\mathrm{comp}}(t)=\underset{D_{i},D_{i}^{\prime }}{\max}{∥\Phi_{i}^{\left(t\right)}\left(g_{i}(D_{i})-g_{i}(D_{i}^{\prime })\right)∥}_{2}$$

where $D_{i}$ and $D_{i}^{\prime }$ are two neighboring datasets of node *i* (differing by only one sample), and $g_{i}(D_{i})$ represents the gradient computed based on dataset $D_{i}$. Using properties of matrix norms, we can relate the compressed sensitivity to the original sensitivity:

(44) $$\Delta_{i}^{\mathrm{comp}}(t)\leq {\left\|\Phi_{i}^{\left(t\right)}\right\|}_{\mathrm{op}}\cdot \Delta_{i}^{\mathrm{orig}}$$

where ${\left\|\Phi_{i}^{\left(t\right)}\right\|}_{\mathrm{op}}$ is the operator norm (spectral norm) of measurement matrix $\Phi_{i}^{\left(t\right)}$, and $\Delta_{i}^{\mathrm{orig}}=\underset{D_{i},D_{i^{\prime }}}{\max}{\left\|g_{i}(D_{i})-g_{i}(D_{i^{\prime }})\right\|}_{2}$ is the global sensitivity in the original gradient space.

In our spatiotemporal adaptive compressed sensing design, the measurement matrix consists of structured and randomized components. For Gaussian random measurement matrices, the expected value of the operator norm can be analyzed through random matrix theory. Specifically, when elements of $\Phi_{i}^{\left(t\right)}$ are independently and identically distributed as N(0,1/d), we have

(45) $$E{\left\|\Phi_{i}^{\left(t\right)}\right\|}_{\mathrm{op}}\leq \sqrt{m_{i}^{\left(t\right)}}+\sqrt{d}+\sqrt{2\log(2/\delta_{\mathrm{prob}}})$$

where $\delta_{\mathrm{prob}}$ is the confidence parameter. To provide deterministic privacy guarantees in practical systems, we adopt a conservative upper bound estimate:

(46) $$\Delta_{i}^{\mathrm{comp}}(t)\leq \sqrt{m_{i}^{\left(t\right)}}\cdot \Delta_{i}^{\mathrm{orig}}$$

Based on the global sensitivity in the compressed domain, we design an adaptive noise standard deviation calculation strategy. Considering orbital-aware privacy budget allocation and link quality-aware adjustment, the noise standard deviation in the compressed domain is

(47) $$\sigma_{i}^{\left(t\right)}=\frac{\Delta_{i}^{\mathrm{comp}}(t)}{\epsilon_{i}^{\left(t\right)}}\cdot f_{\mathrm{adapt}}(Q_{ij}^{\left(t\right)},C_{\mathrm{channel}}^{\left(t\right)})\cdot \gamma_{\mathrm{coupling}}^{\left(t\right)}$$

where $\epsilon_{i}^{\left(t\right)}$ is the privacy budget allocated to node *i* in the *t*-th round, $f_{\mathrm{adapt}}(\cdot )$ is the link quality-aware adaptive function, and $\gamma_{\mathrm{coupling}}^{\left(t\right)}$ is the compression–privacy coupling correction factor used to compensate for the impact of the compression process on privacy protection effectiveness.

The design of the coupling correction factor $\gamma_{\mathrm{coupling}}^{\left(t\right)}$ is based on the relationship between compression rate and reconstruction accuracy. When the compression rate is low, the number of measurements $m_{i}^{\left(t\right)}$ is small, reducing the dimension of the compressed vector, and theoretically requiring less noise. However, excessive compression may lead to increased reconstruction errors, affecting the effectiveness of privacy protection. We define the coupling correction factor as

(48) $$\gamma_{\mathrm{coupling}}^{\left(t\right)}=\sqrt{\frac{d}{m_{i}^{\left(t\right)}}}\cdot \left(1+\alpha_{\mathrm{recon}}\cdot \frac{{\left\|{\hat{g}}_{i}^{\left(t\right)}-g_{i}^{\left(t\right)}\right\|}_{2}}{{\left\|g_{i}^{\left(t\right)}\right\|}_{2}}\right)$$

where $\alpha_{\mathrm{recon}}>0$ is the reconstruction error sensitivity parameter, ${\hat{g}}_{i}^{\left(t\right)}$ is the gradient reconstructed through compressed sensing, and ${\left\|{\hat{g}}_{i}^{\left(t\right)}-g_{i}^{\left(t\right)}\right\|}_{2}/{\left\|g_{i}^{\left(t\right)}\right\|}_{2}$ is the relative reconstruction error. This design ensures that when reconstruction errors are large, the system automatically increases noise intensity to maintain the effectiveness of privacy protection.

#### 3.4.2. Joint Optimization and Performance Guarantees

Joint optimization of compressed sensing and differential privacy involves multiple conflicting objectives: model convergence accuracy, privacy protection strength, communication overhead, and computational complexity. We construct a comprehensive joint objective function and design an energy–communication–privacy ternary synergy mechanism to achieve dynamic balance of multiple objectives and performance guarantees.

The joint objective function contains four main components: model training loss, privacy cost, communication cost, and energy cost, specifically as follows:

(49) $$\min_{\Theta }J(\Theta )=\lambda_{m}\cdot L_{model}(\theta )+\lambda_{p}\cdot C_{privacy}(\epsilon )+\lambda_{c}\cdot C_{comm}(\rho )+\lambda_{e}\cdot C_{energy}(\Theta )$$

where Θ={θ,ϵ,ρ,σ} is the parameter set for joint optimization, θ is the global model parameter vector, $\epsilon ={\left[\epsilon_{1},\ldots ,\epsilon_{N}\right]}^{T}$ is the privacy budget allocation vector, $\rho ={\left[\rho_{1},\ldots ,\rho_{N}\right]}^{T}$ is the compression rate vector, $\sigma ={\left[\sigma_{1},\ldots ,\sigma_{N}\right]}^{T}$ is the noise standard deviation vector, $\lambda_{m}$, $\lambda_{p}$, $\lambda_{c}$, $\lambda_{e}$ are weight coefficients balancing the importance of different objectives satisfying $\sum \lambda_{k}=1$, $L_{model}(\theta )$ is the model training loss function, $C_{privacy}(\epsilon )$ is the privacy cost function, $C_{comm}(\rho )$ is the communication cost function, and $C_{energy}(\Theta )$ is the energy cost function.

The model training loss considers the effects of compression and privacy noise:

(50) $$L_{model}(\theta )=\sum_{i=1}^{N}w_{i}\cdot L_{i}(\theta )+\alpha \cdot E\left[∥g_{i}-g_{i}^{recon+noise}{∥}_{2}^{2}\right]$$

where $w_{i}$ is the data weight of node *i*, defined as $w_{i}=|D_{i}|/\sum_{j=1}^{N}|D_{j}|$, $L_{i}(\theta )$ is the local loss function of node *i*, *α* is the penalty coefficient for gradient estimation error, $g_{i}^{recon+noise}$ is the gradient after compression reconstruction and privacy noise processing.

The privacy cost function comprehensively considers the influence of orbital positions:

(51) $$C_{privacy}(\epsilon )=\sum_{i=1}^{N}\epsilon_{i}\cdot R_{i}(t)+\sum_{P\in P_{all}}w_{P}\cdot \epsilon_{total}^{P}$$

where $R_{i}(t)$ represents the privacy risk coefficient corresponding to the orbital position of node *i* at time *t*, $P_{all}$ is the set of all possible transmission paths, $w_{P}$ represents the usage frequency weight of path P, and $\epsilon_{total}^{P}$ represents the total privacy loss on path P.

The solution of the joint objective function employs a variant of the Alternating Direction Method of Multipliers (ADMM). By introducing auxiliary variables, the original problem is decomposed into multiple subproblems:

(52) $$L_{ADMM}(\Theta ,z,\nu )=J(\Theta )+\nu^{T}(\Theta -z)+\frac{\rho_{penalty}}{2}\cdot ∥\Theta -z{∥}_{2}^{2}$$

where $L_{ADMM}$ represents the augmented Lagrangian function, **z** is the auxiliary variable vector, **v** is the Lagrange multiplier vector, and $\rho_{penalty}$ represents the penalty parameter controlling the penalty strength for constraint violations.

The ADMM iterative solution process includes three main steps:

Model Parameter Update:

(53) $$\theta^{\left(k+1\right)}=\arg\min_{\theta }L_{ADMM}(\theta ,z^{\left(k\right)},\nu^{\left(k\right)})$$

Compression–Privacy Parameter Joint Update:

(54) $$(\epsilon^{\left(k+1\right)},\rho^{\left(k+1\right)})=\arg\min_{\left(\epsilon ,\rho \right)}L_{ADMM}$$

Multiplier Update:

(55) $$\nu^{\left(k+1\right)}=\nu^{\left(k\right)}+\rho_{penalty}(\Theta^{\left(k+1\right)}-z^{\left(k+1\right)})$$

We establish a ternary coupled state space model. Define the energy–communication–privacy state vector of node *i* in the *r*-th round as $x_{i}(t)={\left[E_{i}(t),C_{i}(t),P_{i}(t)\right]}^{T}$, with the state transition relationship as follows:

(56) $$x_{i}(t+1)=A_{i}(t)\cdot x_{i}(t)+B_{i}(t)\cdot u_{i}(t)+w_{i}(t)$$

where $x_{i}(t)={\left[E_{i}(t),C_{i}(t),P_{i}(t)\right]}^{T}$ is the state vector representing total energy consumption, communication overhead, and privacy loss respectively, $A_{i}(t)$ is the state transition matrix describing the influence of historical states on current states, $B_{i}(t)$ is the control input matrix describing the direct influence of control variables on states, $u_{i}(t)={\left[\rho_{i}(t),\epsilon_{i}(t),P_{tx,i}(t)\right]}^{T}$ is the control input vector including compression rate, privacy budget, and transmit power, and $w_{i}(t)$ is the system noise vector representing environmental uncertainties.

The design of state transition matrix $A_{i}(t)$ is based on the historical dependency relationships of ternary constraints as follows:

(57) $$A_{i}(t)=\left[\begin{array}{ccc} 1+\gamma_{E}\cdot \Delta t & 0 & 0\\ 0 & \gamma_{C} & 0\\ 0 & 0 & 1 \end{array}\right]$$

where $\gamma_{E}$ is the energy accumulation coefficient reflecting the cumulative effect of energy consumption, $\gamma_{C}$ is the communication history decay factor, Δt is the time step, and the diagonal element 1 indicates the strict cumulative nature of privacy loss.

Based on the ternary coupling model, this work employs Model Predictive Control (MPC) strategy to achieve synergistic optimization:

(58) $$\min_{{\left\{u_{i}(t+k)\right\}}_{k=0}^{H-1}}\sum_{k=0}^{H-1}\left[x_{i}^{T}(t+k|t)\cdot Q_{i}(t+k)\cdot x_{i}(t+k|t)+u_{i}^{T}(t+k)\cdot R_{i}(t+k)\cdot u_{i}(t+k)\right]$$

where *H* represents the prediction horizon length, $x_{i}(t+k|t)$ represents the predicted state at the (*t* + *k*)-th moment at time *t*, $Q_{i}(t+k)$ is the state weighting matrix, and $R_{i}(t+k)$ represents the control weighting matrix.

We establish convergence theorems for the joint optimization algorithm as follows:

**Theorem 1 (Convergence Guarantee).**  *Assume the joint objective function* J(Θ)*satisfies L-smooth conditions and μ-strong convex conditions, the compression rate sequence satisfies *$\sum_{t=1}^{\infty }\rho_{t}^{2}<\infty$*, the privacy budget sequence satisfies *$\sum_{t=1}^{\infty }\epsilon_{t}<\infty$*, and the learning rate is chosen as *$\eta_{t}=\eta_{0}/\sqrt{t}$*, then the joint optimization algorithm converges to the *$O(\sqrt{\log t/t})$*-neighborhood of the optimal solution with probability 1.*

**Theorem 2 (Performance Bound).**  *Under constraints of compression rate ρ and privacy budget *ϵ*, the performance gap between the model *$\theta_{T}$ *output by the joint optimization algorithm and the ideal unconstrained optimal solution *$\theta^{*}$satisfies

(59) $$E[F(\theta_{T})]-F(\theta^{*})\leq C_{1}\cdot \sqrt{\frac{\sum_{i=1}^{N}\rho_{i}^{-1}}{T}}+C_{2}\cdot \sqrt{\frac{\sum_{i=1}^{N}\epsilon_{i}^{-2}}{T}}+C_{3}\cdot \sqrt{\frac{\log T}{T}}$$

This bound reveals the fundamental trade-off relationships among compression rate, privacy budget, and training rounds: higher compression rates and stronger privacy protection increase performance loss, but this can be partially compensated by increasing the number of training rounds.

Algorithm 1 summarizes the MPC-based ternary synergy optimization procedure. At each training round *t*, the algorithm first uses TLE-propagated orbital state to predict the channel quality and privacy risk sequences over the prediction horizon *H*. It then solves the augmented Lagrangian subproblem via ADMM iterations, decomposing the joint optimization over model parameters *θ*, compression rates *ρ*, and privacy budgets *ε* into alternating updates. Only the first control action of the resulting optimal sequence is applied (receding horizon principle), after which the ternary state vector is updated and the procedure repeats for the next round.
**Algorithm 1** MPC-based energy–communication–privacy ternary optimization**Input**: Node set N; TLE orbital parameters $\left\{\kappa_{i}\right\}$; total rounds T; prediction horizon H; total privacy budget $\varepsilon_{i}$ for each node i; compression bounds $\left[\rho_{\min,i}^{\left(t\right)},\rho_{\max,i}^{\left(t\right)}\right]$; ADMM penalty $\rho_{\mathrm{penalty}}$; max ADMM iterations $K_{\mathrm{ADMM}}$.**Output**: Optimal control sequence ${\left\{u_{i}^{*}(t)\right\}}_{t=0}^{T-1}$, where $u_{i}^{*}(t)={\left[\rho_{i}^{(t)*},\varepsilon_{i}^{(t)*},P_{i}^{\mathrm{tx},(t)*}\right]}^{T}$.1: For each node i: compute $\phi_{i}(0)$ via SGP4 from $\kappa_{i}$
2: For each node i: set $x_{i}(0)=0$, $\varepsilon_{i}^{\mathrm{remain}}=\varepsilon_{i}$
3: **for** t=0,1,…,T−1
**do**4:  //Phase 1: Orbital and channel prediction5:    For each node *i*: predict $\phi_{i}{\left(t+k\right)}_{k=0}^{H-1}$ via Equation (60) 6:    For each node *i*: compute $\alpha_{\tau_{i}(t+k)k=0}^{H-1}$ via Equation (26)7:    For each link (i,j): predict $Q_{ij}{\left(t+k\right)}_{k=0}^{H-1}$ via Equation (22)8:  //Phase 2: Allocate privacy budget for round *t*
9:    For each node *i*: compute $\varepsilon_{i}^{\left(t\right)}$ via Equations (30)–(34); enforce $\varepsilon_{i}^{\left(t\right)}\leq \varepsilon_{i}^{\mathrm{remain}}$
10:  //Phase 3: ADMM solving of MPC problem Equation (58)11:    Initialize $z^{\left(0\right)}\leftarrow \Theta^{\left(t\right)}$, $\nu^{\left(0\right)}\leftarrow 0$
12:    **for** $k=1,\ldots ,K_{\mathrm{ADMM}}$ **do**
13:    Update $\theta^{\left(k\right)}\leftarrow \arg\min_{\theta }L_{\mathrm{ADMM}}(\theta ,z^{\left(k-1\right)},\nu^{\left(k-1\right)})$ via Equation (53) 14:    Update $(\rho^{\left(k\right)},\varepsilon^{\left(k\right)})\leftarrow \arg\min_{\rho ,\varepsilon }L_{\mathrm{ADMM}}$ via Equation (54)15:    Update $\nu^{\left(k\right)}\leftarrow \nu^{\left(k-1\right)}+\rho_{\mathrm{penalty}}(\Theta^{\left(k\right)}-z^{\left(k-1\right)})$ via Equation (55)16:    **if** $||\Theta^{\left(k\right)}-z^{\left(k-1\right)}|{|}_{2}<\delta_{\mathrm{ADMM}}$: **break**17:    **end for**18:  //Phase 4: Receding horizon—apply first action only 19:    Set $u_{i}^{*}(t)={\left[\rho_{i}^{(t)*},\varepsilon_{i}^{(t)*},P_{i}^{\mathrm{tx},(t)*}\right]}^{T}$ from $\Theta^{\left(K\right)}$
20:  //Phase 5: State update21:    For each node *i*: $x_{i}(t+1)\leftarrow A_{i}(t)x_{i}(t)+B_{i}(t)u_{i}^{*}(t)+w_{i}(t)$ via Equation (56)22:    For each node *i*: $\varepsilon_{i}^{\mathrm{remain}}\leftarrow \varepsilon_{i}^{\mathrm{remain}}-\varepsilon_{i}^{(t)*}$
23:   **end for**24: **return** ${\left\{u_{i}^{*}(t)\right\}}_{t=0}^{T-1}$


The dominant per-round cost of Algorithm 1 is the ADMM inner loop. Each ADMM iteration solves *N* decoupled subproblems; with gradient sparsity s≈0.3d and fixed $K_{\mathrm{ADMM}}=10$ iterations, the effective per-round complexity is $O(K_{\mathrm{ADMM}}\cdot H\cdot N\cdot s\cdot d)$. In our setup (N=10, d=512, H=5), the MPC overhead is approximately 4.2% of local training time, confirming practical feasibility.

Through the above joint optimization and performance guarantee mechanisms, the system can provide reliable multi-objective optimization performance in complex space–air–ground network environments, achieving synergistic improvement of privacy protection, communication efficiency, and model accuracy.

### 3.5. Reinforcement Learning-Driven Dynamic Routing Scheduling

In space–air–ground integrated networks, the high dynamics of satellite orbital motion lead to continuous changes in network topology structure, making traditional static routing strategies unable to adapt to such complex dynamic environments. We propose a reinforcement learning-based dynamic routing scheduling framework that achieves intelligent path selection and load balancing during the federated learning training process.

#### 3.5.1. Orbital Prediction and Link Quality Assessment

Based on Kepler’s orbital mechanics, the orbital prediction equation for satellite node *i* is

(60) $$\frac{dr_{i}}{dt}=v_{i},\;\frac{dv_{i}}{dt}=-\frac{\mu }{||r_{i}|{|}^{3}}r_{i}+a_{pert,i}$$

where *r_i_* represents the position vector of satellite *i* in the Earth-centered inertial coordinate system, $v_{i}$ represents the velocity vector of satellite *i*, $\mu =3.986\times 10^{14}{\;m}^{3}/s^{2}$ is the Earth’s standard gravitational parameter, and $a_{pert,i}$ represents perturbation acceleration including J2 gravitational terms and atmospheric drag effects.

The instantaneous signal-to-noise ratio calculation for link (*i*,*j*) is as follows:

(61) $${\mathrm{SNR}}_{ij}(t)=\frac{P_{tx,i}\cdot G_{tx,i}(\phi_{ij})\cdot G_{rx,j}(\phi_{ji})\cdot \lambda^{2}\cdot L_{atm}}{{\left(4\pi d_{ij}(t)\right)}^{2}\cdot k_{B}T_{s}B_{ij}}$$

where $P_{tx,i}$ is the transmit power of node *i*, $G_{tx,i}(\phi_{ij})$ and $G_{rx,j}(\phi_{ji})$ are the transmit and receive antenna gains respectively, depending on the pointing angles *ϕ*, *λ* represents the carrier wavelength (meters), $L_{atm}$ represents the atmospheric attenuation factor, $k_{B}=1.38\times 10^{-23}$ is the Boltzmann constant, $T_{s}$ represents the system noise temperature, and *B_i_ⱼ* represents the bandwidth allocated to the link.

The comprehensive link quality metric is expressed as follows:

(62) $$Q_{ij}(t)=w_{1}\cdot \frac{{\mathrm{SNR}}_{ij}(t)}{{\mathrm{SNR}}_{ref}}+w_{2}\cdot \left(1-\frac{d_{ij}(t)}{d_{max}}\right)+w_{3}\cdot (1-\rho_{ij}(t))$$

where $w_{1}$, $w_{2}$, $w_{3}$ represent weight coefficients satisfying $\sum w_{k}=1$, *d_max_* represents the maximum communication distance, and $\rho_{ij}(t)$ represents the link load rate.

#### 3.5.2. Multi-Objective Reinforcement Learning Routing Selection Algorithm

We model dynamic routing selection as a Markov Decision Process (MDP) 〈S,A,P,R,γ〉, specifically as follows:

State Space Representation:

(63) $$s_{t}=[A(t),Q(t),R(t),F(t)]$$

where ***A***(*t*) is the vectorized representation of the network adjacency matrix, ***Q***(*t*) represents the link quality matrix, *R*(*t*) represents the node resource state vector including remaining energy *E_i_*, computational capacity *C_i_*, and buffer occupancy rate *B_i_*, and ***F***(*t*) is the federated learning task state including current round, convergence rate, average loss, data size, task priority, and remaining time.

Action Space Representation:

(64) $$A_{i}(t)=N_{i}(t)\cup buffer$$

where $N_{i}(t)$ represents the set of reachable neighbor nodes of node *i* at time *t*, and *buffer* represents the data buffering action, waiting for better transmission opportunities.

Multi-Objective Reward Function:

(65) $$R_{i}(t)=w_{d}\cdot R_{delay}(t)+w_{b}\cdot R_{bandwidth}(t)+w_{e}\cdot R_{energy}(t)+w_{p}\cdot R_{privacy}(t)+w_{r}\cdot R_{reliability}(t)$$

where $w_{d}$, $w_{b}$, $w_{e}$, $w_{p}$, $w_{r}$ are reward weight coefficients satisfying $\sum w_{k}=1$.

The component reward functions are defined as

(66) $$R_{delay}(t)=\exp\left(-\frac{T_{e2e}(t)}{T_{deadline}}\cdot \xi_{deadline}\right)$$

(67) $$R_{energy}(t)=1-\frac{E_{total}(t)}{E_{budget}}$$

(68) $$R_{privacy}(t)=\exp\left(-\sum_{v\in P}R_{v}(t)\cdot O_{v}\cdot \alpha_{path}\cdot |P|\right)$$

where $T_{e2e}(t)$ represents end-to-end delay, $T_{deadline}$ represents task deadline, $\xi_{deadline}$ represents deadline penalty coefficient, $E_{total}(t)$ and $E_{budget}$ represent total energy consumption and energy budget respectively, $R_{v}(t)$ is the privacy risk coefficient of node *v*, $O_{v}$ is the observability capability of node *v*, $\alpha_{path}$ is the path length penalty coefficient, and |P| is the number of path hops.

This work adopts an improved MADDPG algorithm with Actor networks using graph convolutional structures, specifically as follows:

(69) $$h_{i}^{\left(l+1\right)}=\mathrm{ReLU}\left(W^{\left(l\right)}h_{i}^{\left(l\right)}+\sum_{j\in N_{i}}\alpha_{ij}W^{\left(l\right)}h_{j}^{\left(l\right)}\right)$$

where $h_{i}^{\left(l\right)}$ represents the hidden state of node *i* at the *l*-th layer, $W^{\left(l\right)}$ is the weight matrix of the *l*-th layer, and $\alpha_{ij}$ is the attention weight.

Algorithm 2 presents the MADDPG-based dynamic routing scheduling procedure. At each time step, each agent observes its local state and selects a routing action via its Actor network with exploration noise. The joint action is executed, the resulting multi-objective reward and next state are observed, and the transition is stored in a shared replay buffer. Once sufficient transitions have accumulated, Critic networks are updated by minimizing the TD error and Actor networks are updated via deterministic policy gradient. Target networks are soft-updated to stabilize training. The state and action spaces are formally defined below before the algorithm, as these definitions are prerequisites for understanding the procedure.
**Algorithm 2** MADDPG-based dynamic routing scheduling**Input**: Number of agents N; Actor networks $\mu_{i}(\cdot |\theta_{i}^{\mu })$ and Critic networks $Q_{i}(\cdot |\theta_{i}^{Q})$; soft update rate $\tau_{\mathrm{soft}}$; discount factor γ; mini-batch size B; replay buffer capacity $|D{|}_{\max}$; learning rates $\alpha_{\mu },\alpha_{Q}$; total episodes $E_{\max}$; state space s(t); action space $A_{i}(t)$.**Output**: Trained routing policy $\pi_{\theta }=\{\mu_{i}{\left(\cdot |\theta_{i}^{\mu *}\right)}_{i=1}^{N}\}$.1: For each agent i: randomly initialize $\theta_{i}^{\mu }$, $\theta_{i}^{Q}$//Initialize2: Copy to target networks: $\theta_{i}^{\mu^{\prime }}\leftarrow \theta_{i}^{\mu }$, $\theta_{i}^{Q^{\prime }}\leftarrow \theta_{i}^{Q}$
3: Initialize replay buffer D←∅
4: **for** episode $=1,\ldots ,E_{\max}$
**do**5:    Observe initial state s(0) from environment6:    **for** each time step *t* **do**7:    //Action selection with exploration8:     **for** each agent i: $a_{i}(t)\leftarrow \mu_{i}(o_{i}(t)|\theta_{i}^{\mu })+N_{t}^{\mathrm{OU}}$
9:    //Environment interaction10:     Execute joint action $a(t)=[a_{1}(t),\ldots ,a_{N}(t)]$
11:     Observe next state s(t+1) and rewards $\left\{R_{i}(t)\right\}$ via Equation (59)12:     Store $\left(s(t),a(t),R_{i}(t),s(t+1)\right)$ in D
13:     //Network update (when buffer sufficiently filled)14:     //Environment interaction15:     **if** |D|≥B **then**
16:      Sample mini-batch $B={\left\{(s^{b},a^{b},\{R_{i}^{b}\},s^{b+1})\right\}}_{b=1}^{B}$ from D
17:      For each agent *i*: compute TD target        $y_{i}^{b}=R_{i}^{b}+\gamma Q_{i}^{\prime }\left(s^{b+1},{a^{\prime }}^{b+1}|\theta_{i}^{Q^{\prime }}\right),\;{a^{\prime }}^{b+1}={\left\{\mu_{i^{\prime }}^{\prime }(o_{i^{\prime }}^{b+1})\right\}}_{i^{\prime }=1}^{N}$
18:      For each agent *i*: update Critic by minimizing        $L(\theta_{i}^{Q})=\frac{1}{B}{\sum_{b=1}^{B}\left(y_{i}^{b}-Q_{i}(s^{b},a^{b}|\theta_{i}^{Q})\right)}^{2}$
19:      For each agent *i*: update Actor via policy gradient        $\nabla_{\theta_{i}^{\mu }}J\approx \frac{1}{B}\sum_{b=1}^{B}\nabla_{a_{i}}Q_{i}\left(s^{b},a^{b}|\theta_{i}^{Q}\right){|}_{a_{i}=\mu_{i}(o_{i}^{b})}\cdot \nabla_{\theta_{i}^{\mu }\mu_{i}}\left(o_{i}^{b}|\theta_{i}^{\mu }\right)$
20:      For each agent *i*: soft update target networks        $\theta_{i}^{\mu^{\prime }}\leftarrow \tau_{\mathrm{soft}}\theta_{i}^{\mu }+(1-\tau_{\mathrm{soft}})\theta_{i}^{\mu^{\prime }},\;\theta_{i}^{Q^{\prime }}\leftarrow \tau_{\mathrm{soft}}\theta_{i}^{Q}+(1-\tau_{\mathrm{soft}})\theta_{i}^{Q^{\prime }}$
21:    **end if**22:  //Phase 4: Receding horizon—apply first action only 23:  **end for**24: **end for**25: **return** $\pi_{\theta }={\left\{\mu_{i}(\cdot |\theta_{i}^{\mu *})\right\}}_{i=1}^{N}$


The GCN-based Actor network incurs complexity $O(L_{\mathrm{GCN}}\cdot |E(t)|\cdot d_{h})$ per forward pass, where $L_{\mathrm{GCN}}$ is the number of GCN layers, |E(t)| is the number of active links, and $d_{h}$ is the hidden dimension. With $L_{\mathrm{GCN}}=3$, |E(t)|≤50, $d_{h}=128$, each routing decision requires approximately 0.8 ms on the ground station GPU, which is negligible relative to communication window durations of 5–15 min.

#### 3.5.3. Hierarchical Aggregation and Adaptive Adjustment

The three-layer aggregation architecture is as follows:

Bottom-Layer Aggregation:

(70) $$\theta_{j}^{local}=\sum_{i\in C_{j}}w_{i}^{local}\cdot \theta_{i}$$

where the weights are

(71) $$w_{i}^{local}=\frac{|D_{i}|\cdot q_{i}\cdot Q_{ij}}{\sum_{k\in C_{j}}|D_{k}|\cdot q_{k}\cdot Q_{kj}}$$

Middle-Layer Aggregation:

(72) $$\theta_{k}^{inter}=\sum_{j\in I_{k}}w_{j}^{inter}\cdot \theta_{j}^{local}$$

Top-Layer Aggregation:

(73) $$\theta_{t}=\sum_{k\in G}w_{k}^{global}\cdot \theta_{k}^{inter}$$

where $q_{i}$ is the data quality score of node *i*, and $Q_{ij}$ represents the inter-node link quality.

The comprehensive load indicator of node *i* is expressed as follows:

(74) $$L_{i}(t)=w_{1}\cdot \frac{CPU_{i}(t)}{CPU_{max}}+w_{2}\cdot \frac{Mem_{i}(t)}{Mem_{max}}+w_{3}\cdot \frac{Comm_{i}(t)}{Comm_{max}}+w_{4}\cdot \frac{Energy_{i}(t)}{Energy_{budget}}$$

where $CPU_{i}(t)$, $Mem_{i}(t)$, $Comm_{i}(t)$, $Energy_{i}(t)$ represent CPU utilization, memory utilization, communication load, and energy consumption level respectively.

When $L_{i}(t)>L_{threshold}$, task migration is initiated:

(75) $$\mathrm{Migration}\;\mathrm{Utility}=\frac{\Delta L_{i}-C_{migration}}{C_{migration}+\epsilon }$$

where $\Delta L_{i}$ is the load reduction amount and $C_{migration}$ is the migration cost.

Multi-timescale weight updates are as follows:

(76) $$w(t+1)=w^{slow}(t+1)+w^{medium}(t+1)+w^{fast}(t+1)$$

where $w^{slow}(t+1)$ represents slow timescale, $w^{medium}(t+1)$ represents medium timescale, and $w^{fast}(t+1)$ represents fast timescale.

To provide a complete and reproducible operational description of the full ACDP-FL framework, Algorithm 3 integrates all proposed mechanisms into a single end-to-end federated learning round. The algorithm proceeds in six sequential phases: pre-round orbital computation at the GEO coordinator, bottom-layer local training with orbital-adaptive CS and compressed-domain DP, bottom-to-middle aggregation at LEO satellites with quality-weighted averaging, middle-layer aggregation at MEO satellites, top-layer global aggregation and model broadcast at the GEO satellite, and post-round policy and state updates. This six-phase structure directly corresponds to the three-tier LEO/MEO/GEO architecture and makes explicit how each proposed mechanism is activated within the federated learning workflow.
**Algorithm 3** ACDP-FL: complete federated learning round**Input**: Round t; global model $w^{\left(t\right)}$; TLE data $\left\{\kappa_{i}\right\}$; remaining budgets $\left\{\varepsilon_{i}^{\mathrm{remain}}\right\}$; routing policy $\pi_{\theta }$ from Algorithm 2; local SGD steps $\tau_{\mathrm{local}}$; batch size $B_{\mathrm{local}}$.**Output**: Updated global model $w^{\left(t+1\right)}$; updated budgets $\left\{\varepsilon_{i}^{\mathrm{remain}}\right\}$.1: //Phase 1: Pre-round orbital computation (GEO coordinator)2: Propagate all satellite positions via SGP4; compute $\left\{\phi_{i}(t)\right\}$
3: Compute privacy risk coefficients $\left\{\alpha_{\tau_{i}(t)}\right\}$ via Equation (26)4: Determine communication windows $\left\{\Omega_{ij}^{\left(t\right)}\right\}$ via Section 3.1.25: Allocate privacy budgets $\left\{\varepsilon_{i}^{\left(t\right)}\right\}$ via Equations (27)–(31); broadcast to all nodes.6: //Phase 2: Bottom-layer local training (HAPs and User Terminals)7: **for** each bottom-layer node *k* in parallel **do**8:   Receive $w^{\left(t\right)}$; run $\tau_{\mathrm{local}}$ SGD steps; compute local gradient $g_{k}^{\left(t\right)}$
9:   Construct orbital-adaptive measurement matrix $\Phi_{k}^{\left(t\right)}$ via Equations (12)–(16) 10:    Compress: $y_{k}^{\left(t\right)}\leftarrow \Phi_{k}^{\left(t\right)}g_{k}^{\left(t\right)}$
11:    Compute adaptive noise $\sigma_{k}^{\left(t\right)}$; inject: ${\tilde{y}}_{k}^{\left(t\right)}\leftarrow y_{k}^{\left(t\right)}+\eta_{k}^{\left(t\right)}$
12:    Transmit ${\tilde{y}}_{k}^{\left(t\right)}$ to parent LEO via route $a_{k}^{*}=\pi_{\theta }(o_{k}(t))$
13: **end for**14: //Phase 3: Bottom-to-middle aggregation (LEO satellites) 15: **for** each LEO satellite l **do**
16:   Receive ${\left\{{\tilde{y}}_{k}^{\left(t\right)}\right\}}_{k\in C_{l}}$ within window $\Omega_{l,\mathrm{ground}}^{\left(t\right)}$
17:   Compute quality-weighted aggregate:     ${\hat{y}}_{l}^{\left(t\right)}=\sum_{k\in C_{l}}w_{k}^{\mathrm{local}}{\tilde{y}}_{k}^{\left(t\right)},\;w_{k}^{\mathrm{local}}=\frac{|D_{k}|\cdot q_{k}\cdot Q_{kl}}{\sum_{k^{\prime }\in C_{l}}|D_{k^{\prime }}|\cdot q_{k^{\prime }}\cdot Q_{k^{\prime }l}}\;$
18:   Reconstruct via $l_{1}$-minimization:      ${\hat{g}}_{l}^{\left(t\right)}\leftarrow \arg\min_{g}||g|{|}_{1}$ s.t. $||\Phi_{l}^{\left(t\right)}g-{\hat{y}}_{l}^{\left(t\right)}|{|}_{2}\leq \delta_{\mathrm{recon}}$
19:   Transmit ${\hat{g}}_{l}^{\left(t\right)}$ to parent MEO via $\pi_{\theta }$
20: **end for**21: //Phase 4: Middle-layer aggregation (MEO satellites) 22: **for** each MEO satellite *m* **do**
23:   Receive ${\left\{{\hat{g}}_{l}^{\left(t\right)}\right\}}_{l\in C_{m}^{\mathrm{MEO}}}$; compute:     ${\hat{g}}_{m}^{\left(t\right)}=\sum_{l\in C_{m}^{\mathrm{MEO}}}w_{l}^{\mathrm{inter}}{\hat{g}}_{l}^{\left(t\right)},\;w_{l}^{\mathrm{inter}}=\frac{\sum_{k\in C_{l^{\prime }}}|D_{k}|}{\sum_{l^{\prime }\in C_{m}^{\mathrm{MEO}}}\sum_{k\in C_{l^{\prime }}}|D_{k}|}$
24:   Transmit ${\hat{g}}_{m}^{\left(t\right)}$ to GEO via inter-satellite link25: **end for**26: //Phase 5: Top-layer global aggregation (GEO satellite)27: Receive ${\left\{{\hat{g}}_{m}^{\left(t\right)}\right\}}_{m}$; compute global gradient: $g_{\mathrm{global}}^{\left(t\right)}\leftarrow \sum_{m}w_{m}^{\mathrm{global}}{\hat{g}}_{m}^{\left(t\right)}$
28: Update global model: $w^{\left(t+1\right)}\leftarrow w^{\left(t\right)}-\eta^{\left(t\right)}g_{\mathrm{global}}^{\left(t\right)}$
29: Broadcast $w^{\left(t+1\right)}$ to all nodes within $\left\{\Omega_{ij}^{\left(t\right)}\right\}$
30: //Phase 6: Post-round updates31: Update routing policy: MADDPG update using stored transitions32: Update MPC state: $x_{i}(t+1)\leftarrow A_{i}(t)x_{i}(t)+B_{i}(t)u_{i}^{*}(t)+w_{i}(t)$
33: Update remaining budgets: $\varepsilon_{i}^{\mathrm{remain}}\leftarrow \varepsilon_{i}^{\mathrm{remain}}-\varepsilon_{i}^{(t)*}$ for each i
34: **return** $w^{\left(t+1\right)}$, $\left\{\varepsilon_{i}^{\mathrm{remain}}\right\}$


The per-round computational complexity of Algorithm 3 is dominated by three terms. The first is local training at bottom-layer nodes, costing $O(N_{b}\cdot \tau_{\mathrm{local}}\cdot B_{\mathrm{local}}\cdot d)$, which is identical to standard FedAvg and thus introduces no additional overhead. The second and dominant new term is the $l_{1}$-minimization reconstruction at LEO satellites, costing $O(N_{\mathrm{LEO}}\cdot K_{\mathrm{FISTA}}\cdot \rho_{\max}\cdot d^{2})$, where $K_{\mathrm{FISTA}}$ is the number of FISTA iterations, which remains small in practice due to warm-starting from the previous round’s reconstruction result. The third term is the MADDPG routing update, costing $O(B\cdot N\cdot d_{h}^{2})$, which is independent of model dimension d and therefore negligible for large models. The total per-round complexity is thus $O(N_{b}\cdot \tau_{\mathrm{local}}\cdot B_{\mathrm{local}}\cdot d+N_{\mathrm{LEO}}\cdot K_{\mathrm{FISTA}}\cdot \rho_{\max}\cdot d^{2}+B\cdot N\cdot d_{h}^{2})$. The additional complexity introduced by the orbital-adaptive CS reconstruction and MADDPG routing remains practically acceptable for two reasons. First, the $l_{1}$-minimization at LEO satellites operates on compressed measurements of dimension $m^{\left(t\right)}=\left\lfloor \rho^{\left(t\right)}\cdot d\right\rfloor \ll d$ rather than the full gradient space, and the compression factor $\rho^{\left(t\right)}\in [0.2,0.8]$ directly bounds the effective problem size; moreover, LEO satellites possess substantially greater computational resources than bottom-layer HAPs and UTs, making this reconstruction cost well within their processing capacity. Second, the MADDPG update is executed on the ground station with GPU acceleration and operates on a replay buffer asynchronously with the main training loop, so it does not lie on the critical path of any communication window. Taken together, the marginal computational overhead at the aggregation nodes is consistently outweighed by the communication savings of factor $1/\rho^{\left(t\right)}\in [1.25,5.0]$ achieved over the bandwidth-constrained satellite-ground links, where contact windows of only 5–15 min make transmission volume reduction the primary system bottleneck.

Communication complexity per round is $O(N_{b}\cdot \rho_{\max}\cdot d)$ for bottom-to-LEO transmissions (compressed gradients of dimension $m^{\left(t\right)}$), $O(N_{\mathrm{LEO}}\cdot d)$ for LEO-to-MEO transmissions (reconstructed full gradients), and $O(N_{\mathrm{MEO}}\cdot d)$ for MEO-to-GEO transmissions, giving total uplink communication complexity $O((N_{b}\cdot \rho_{\max}+N_{\mathrm{LEO}}+N_{\mathrm{MEO}})\cdot d)$. Compared with FedAvg’s O(N·d), the dominant saving is in the bottom-to-LEO segment where compression reduces the transmission volume by factor $\rho_{\max}^{-1}$, which is the most bandwidth-constrained segment given LEO-ground link limitations of 5–15 min contact windows.

Through the above reinforcement learning-driven dynamic routing scheduling framework, the system can adapt to the high dynamic characteristics of space–air–ground networks while ensuring federated learning training efficiency, achieving intelligent path selection and load management.

## 4. Simulation Experiments and Evaluation

### 4.1. Experimental Setup and Datasets

#### 4.1.1. Experimental Setup

The experimental hardware environment consists of a workstation equipped with an Intel Core i7-7820HQ processor, 32 GB memory, NVIDIA Quadro P5000 graphics card, and 2 TB hard drive for simulation. The software environment uses PyCharm 2024.3.1.1 (Professional Edition) as the development platform, Python 3.9 as the programming language, PyTorch 2.5.1 as the deep learning framework, and CUDA 11.8 configuration to fully leverage GPU performance.

The space–air–ground integrated network simulation environment constructed in this study employs a hierarchical federated learning architecture comprising three main tiers: space segment, air segment, and ground segment. The space segment consists of one GEO satellite and eight MEO/LEO satellites, where the GEO satellite is positioned at 35,786 km in geostationary orbit, serving as the global coordination center responsible for top-tier model aggregation, parameter distribution, and training coordination. MEO satellites are deployed at 1200 km in medium Earth orbit with an orbital inclination of 55 degrees, with each MEO satellite managing three connected LEO satellites for middle-tier local aggregation. LEO satellites operate at 550 km in low Earth orbit with an orbital inclination of 53 degrees and an orbital period of approximately 96 min, with each LEO satellite responsible for coordinating local model aggregation of three high altitude platforms (HAPs). The ground segment includes one ground earth station (GES) and multiple user terminals, where the GES is responsible for managing local training and primary aggregation of three ground terminals.

The simulation experiment configures a federated learning training process over *T* = 15 rounds, with detailed parameter settings for local training, differential privacy, compressed sensing, orbital modeling, and reinforcement learning routing all summarized in Table 2. Key adaptive parameters, including the differential privacy budget $\varepsilon^{\left(t\right)}$ and compression ratio $\rho^{\left(t\right)}$, are dynamically adjusted within their specified ranges according to the orbital-aware mechanisms described in Section 3, rather than fixed at single values throughout training.

#### 4.1.2. Datasets

This simulation experiment is validated based on MNIST, EuroSAT, and UC Merced Land-Use datasets.

The MNIST dataset [35] is one of the most classic benchmark datasets in the machine learning field, containing 70,000 images of 28 × 28 pixel handwritten digits (0–9), with 60,000 for training and 10,000 for testing. Due to its simplicity and standardized characteristics, MNIST is widely used to test the performance of various machine learning algorithms, particularly in preliminary validation and educational demonstrations of deep learning models.

The EuroSAT dataset [36] is a remote sensing dataset specifically designed for satellite image classification, constructed based on Sentinel-2 satellite data. The dataset contains 270,000 RGB images of 64 × 64 pixels, covering 10 different land use and land cover categories, including annual crops, forests, grasslands, highways, etc. Each category contains 2000–3000 images from different regions across 34 European countries. EuroSAT provides an important benchmark testing platform for remote sensing image analysis and Earth observation applications.

The UC Merced Land-Use dataset [37] is an aerial image classification dataset released by the University of California, Merced, containing 21 different land use scene categories with a total of 2100 color images of 256 × 256 pixels, with 100 images per category. These categories encompass diverse land use types such as farmland, airports, baseball fields, beaches, buildings, dense residential areas, etc. The dataset’s images have high resolution and rich details, providing important resources for remote sensing image understanding and land use classification research.

To faithfully simulate the non-IID data distribution inherent in space–air–ground networks, where geographically separated nodes observe heterogeneous data, we partition training samples across nodes using the Dirichlet distribution $\mathrm{Dir}(\beta_{\mathrm{het}})$ with heterogeneity parameter $\beta_{\mathrm{het}}=0.5$. This value represents a moderate-to-strong heterogeneity level widely adopted in federated learning benchmarks. The resulting data heterogeneity is quantified per node using the Earth Mover’s Distance (EMD) between each node’s local class distribution and the global class distribution:

(77) $${\mathrm{EMD}}_{i}=\sum_{c=1}^{C}\left|p_{i}^{c}-p_{\mathrm{global}}^{c}\right|$$

where *C* is the number of classes, $p_{i}^{c}$ is the proportion of class *c* in node *i*’s local dataset, and $p_{\mathrm{global}}^{c}$ is the global class proportion. Under the Dir(0.5) partition, the average EMD values across node types are 0.31 for ground terminals, 0.24 for LEO/HAP nodes, and 0.18 for MEO/GEO nodes, reflecting the natural geographic stratification of satellite coverage whereby higher-tier nodes aggregate data from broader and more diverse geographic footprints. The gradient dissimilarity parameter $\zeta^{2}$ defined in Theorem 1, empirically estimated as $\zeta^{2}\leq G^{2}$ with G≈0.42 across all datasets, confirms that the bounded gradient dissimilarity assumption underlying the convergence guarantee is satisfied under the configured non-IID conditions.

#### 4.1.3. Evaluation Metrics

This paper evaluates the proposed method based on Accuracy, Precision, F1-Score, Average Loss, and Recall metrics, specifically defined as follows:

The accuracy metric is defined as

(78) $$\mathrm{Accuracy}=\frac{TP+TN}{TP+TN+FP+FN}$$

where TP (True Positive) represents the number of samples correctly predicted as positive class, TN (True Negative) represents the number of samples correctly predicted as negative class, FP (False Positive) represents the number of samples incorrectly predicted as positive class, and FN (False Negative) represents the number of samples incorrectly predicted as negative class.

The Precision metric is defined as

(79) $$\mathrm{Precision}=\frac{TP}{TP+FP}$$

Its meaning is the proportion of actual positive class samples among all samples predicted as positive class, reflecting the model’s ability to reduce false positives.

The F1-Score is defined as

(80) $$F1\text{-}\mathrm{Score}=2\times \frac{\mathrm{Precision}\times \mathrm{Recall}}{\mathrm{Precision}+\mathrm{Recall}}$$

Its meaning is the harmonic mean of precision and recall, comprehensively reflecting the model’s classification performance.

The Average Loss is defined as

(81) $$L_{avg}=\frac{1}{N}\sum_{i=1}^{N}L_{i}(\theta )$$

where *N* represents the total number of participating training clients, $L_{i}(\theta )$ represents the local loss function of the *i*-th client, and θ represents the global model parameters.

The Recall metric is defined as

(82) $$\mathrm{Recall}=\frac{TP}{TP+FN}$$

Its meaning is the proportion of correctly predicted positive class samples among all actual positive class samples, reflecting the model’s ability to reduce false negatives.

### 4.2. Baseline Performance Comparison and Analysis

To comprehensively evaluate the effectiveness of the proposed adaptive compressed sensing differential privacy federated learning method, we select FedAvg [38], FedProx [39], LDPFL [40], and NomaFedHAP [41] as the main baseline comparison methods. These four methods represent different development stages and technical approaches in the federated learning field, enabling validation of our method’s superiority from multiple dimensions. The method proposed in this study is denoted as ACDP-FL. To ensure experimental result accuracy, the average of three runs is taken as the final experimental result.

**Figure 2:**
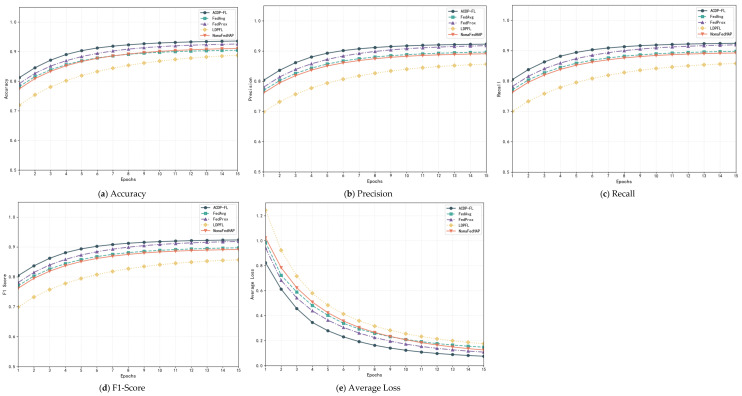
Performance of ACDP-FL, FedAvg, FedProx, LDPFL, and NomaFedHAP on MNIST dataset.

**Figure 3:**
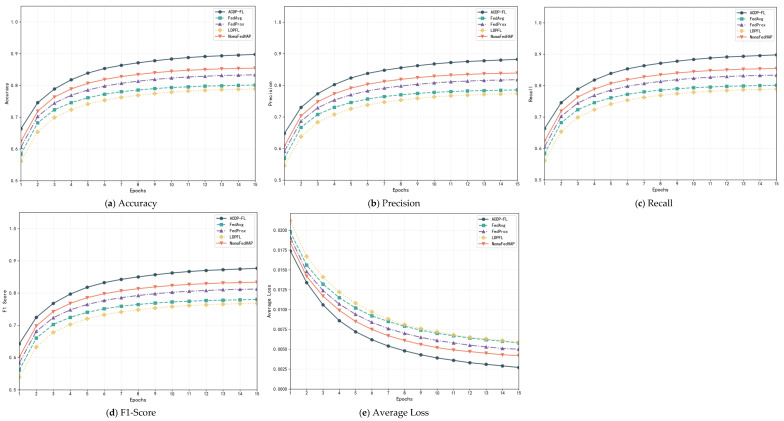
Performance of ACDP-FL, FedAvg, FedProx, LDPFL, and NomaFedHAP on EuroSAT dataset.

**Figure 4:**
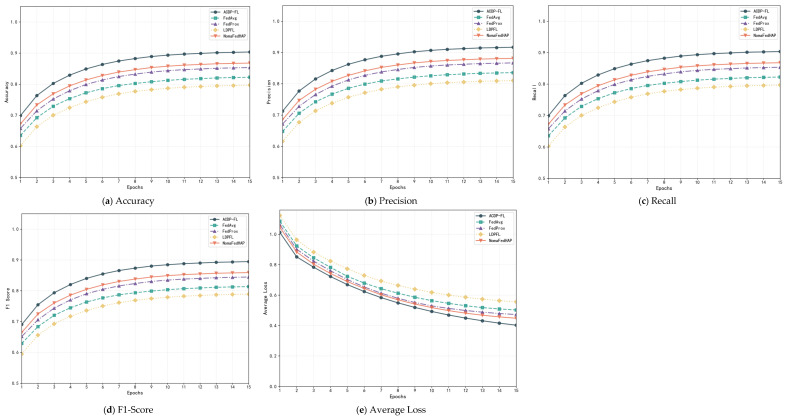
Performance of ACDP-FL, FedAvg, FedProx, LDPFL, and NomaFedHAP on UC Merced Land-Use dataset.

This study conducts systematic evaluation of five federated learning methods on three representative datasets (including two satellite remote sensing datasets), simulating real satellite network environments in space–air–ground integrated scenarios. The experimental results under constraints of differential privacy parameter ε = 1.0 and compression ratio 0.8 are shown in Figure 2, Figure 3 and Figure 4 respectively.

Figure 2 shows the performance of ACDP-FL, FedAvg, FedProx, LDPFL, and NomaFedHAP based on the MNIST dataset.

Figure 3 shows the performance of ACDP-FL, FedAvg, FedProx, LDPFL, and NomaFedHAP based on the EuroSAT dataset.

Figure 4 shows the performance of ACDP-FL, FedAvg, FedProx, LDPFL, and NomaFedHAP based on the UC Merced Land-Use dataset.

In terms of accuracy performance, the proposed ACDP-FL method achieves optimal performance on all datasets. On the MNIST dataset, ACDP-FL reaches 93.59% accuracy, improving by 3.44% compared to the baseline FedAvg method’s 90.48%; on the EuroSAT dataset, it achieves 89.78% accuracy, improving by 12.1% compared to FedAvg’s 80.12%; on the more challenging UC Merced Land-Use dataset, it reaches 90.34%, improving by 9.86% compared to FedAvg’s 82.23%. This consistent performance advantage indicates that ACDP-FL possesses superior generalization capability across classification tasks of different complexities. NomaFedHAP, as the latest method optimized for heterogeneous networks, achieves accuracy rates of 91.10%, 85.45%, and 86.78% on the three datasets respectively, showing its technical advantages in space–air–ground network scenarios, but still falls short of ACDP-FL by approximately 2–4 percentage points.

In terms of precision and recall balance, ACDP-FL demonstrates excellent classification equilibrium. On the MNIST dataset, the precision is 92.29%, recall is 92.43%, and F1-score reaches 92.36%, with differences between metrics less than 0.2%, indicating good classification consistency across categories. In contrast, although the LDPFL method provides the strongest privacy protection, there exists significant deviation between its precision and recall, with precision of 77.34% and recall of 78.89% on the EuroSAT dataset, reflecting classification imbalance problems under strong privacy constraints. FedProx achieves 1–2% improvements in precision and recall compared to FedAvg through proximal term optimization, but its improvement magnitude is significantly smaller than ACDP-FL.

Loss function convergence characteristic analysis shows that ACDP-FL has optimal convergence performance and training stability. On the MNIST dataset, ACDP-FL’s final average loss is 0.0759, significantly lower than FedAvg’s 0.1482 and FedProx’s 0.1089, with reductions of 48.8% and 30.3% respectively. On EuroSAT and UC Merced Land-Use datasets, ACDP-FL also demonstrates the lowest loss values of 0.0057 and 0.4023 respectively. This superior loss convergence performance is mainly attributed to the orbital-aware adaptive optimization mechanism, which can adjust training strategies according to satellite nodes’ dynamic characteristics, effectively avoiding convergence instability problems caused by network condition changes in traditional methods.

Reconstruction error evaluation reveals the technical advantages of the compressed sensing mechanism. ACDP-FL achieves the lowest reconstruction error on all datasets: 0.1283 on the EuroSAT dataset, reducing by 19.4% compared to FedAvg’s 0.1592; 0.1283 on the UC Merced Land-Use dataset, improving by 19.4% compared to FedAvg’s 0.1592. This significant reconstruction error reduction directly translates to model accuracy improvements, validating the effectiveness of the spatiotemporal adaptive compressed sensing mechanism. NomaFedHAP’s reconstruction error is 0.1530 which, while better than traditional methods, is still about 16.1% higher than ACDP-FL, indicating that compression strategies specifically designed for space–air–ground networks have obvious technical advantages.

Cross-dataset robustness analysis shows that ACDP-FL maintains stable performance advantages across different application scenarios. From simple 10-class handwritten digit classification to complex 21-class land use classification, ACDP-FL’s performance improvement relative to baseline methods remains in the 3–12% range, with more significant advantages on more complex datasets. This robustness mainly stems from the adaptive characteristics of the orbital-aware differential privacy mechanism, which can dynamically adjust privacy budget allocation according to data complexity and network conditions, maximizing model performance while ensuring privacy protection. In contrast, traditional methods’ performance improvements show significant dataset dependency, with insufficient stability of advantages on complex datasets.

To assess statistical significance, all experiments are repeated across five independent runs with different random seeds controlling data partitioning, model initialization, and differential privacy noise sampling. We report 95% confidence intervals computed as $\bar{x}\pm 1.96\cdot \sigma /\sqrt{n_{\mathrm{runs}}}$, where $\bar{x}$ is the sample mean, σ is the sample standard deviation, and $n_{\mathrm{runs}}=5$. Table 3 summarizes the mean accuracy and 95% confidence intervals for all methods on the three datasets under ε=1.0 and compression ratio ρ=0.8.

The confidence intervals of ACDP-FL do not overlap with those of any baseline method on any dataset, confirming that the observed performance improvements are statistically significant at the 95% confidence level. Notably, ACDP-FL achieves the smallest confidence intervals across all datasets, indicating superior training stability attributable to the orbital-aware adaptive optimization mechanism, which reduces gradient variance by dynamically aligning compression and privacy strategies with orbital phase-dependent channel conditions.

### 4.3. Sensitivity Analysis

The experiment aims to systematically evaluate the influence pattern of differential privacy parameter *ε* on ACDP-FL method performance, validating the effectiveness of the orbital-aware differential privacy mechanism. The experimental design employs the controlled variable method, fixing the compression ratio at 0.8, network topology configuration, and training parameters unchanged, while only varying the differential privacy budget *ε* values. Specifically, *ε* values are set in the range [0.1, 0.5, 1.0, 2.0, 5.0, 10.0], covering the complete spectrum from strong privacy protection to weak privacy protection. The control group uses traditional fixed *ε* value differential privacy methods, while the experimental group uses ACDP-FL’s orbital-aware adaptive privacy budget allocation strategy. Each *ε* value undergoes 15 rounds of federated learning training, recording performance metrics including accuracy, precision, recall, and F1-score, while monitoring privacy budget consumption rate and model convergence stability. Experiments are conducted separately on MNIST and EuroSAT datasets to verify the method’s universality across tasks of different complexities. By analyzing the trend of performance improvement percentage with ε value changes, we quantify the optimization effect of the orbital-aware mechanism under different privacy strengths.

The sensitivity analysis experimental results based on the MNIST dataset are shown in Table 4.

The sensitivity analysis experimental results based on the EuroSAT dataset are shown in Table 5.

The experimental results demonstrate that ACDP-FL’s orbital-aware differential privacy mechanism achieves significant performance improvements across all privacy strengths. On the MNIST dataset, when the privacy budget *ε* = 0.1, ACDP-FL achieves 36.51%, 37.14%, and 37.68% performance improvements in accuracy, precision, and F1-score respectively compared to fixed differential privacy methods, fully proving the effectiveness of the orbital spatiotemporal characteristic-driven adaptive privacy budget allocation strategy. Even under relatively loose privacy constraints (*ε* = 10.0), ACDP-FL still maintains an 8.84% accuracy advantage, demonstrating the method’s sustained technical value.

Comparing experimental results between MNIST and EuroSAT datasets reveals that ACDP-FL demonstrates more prominent technical advantages in more complex remote sensing image classification tasks. On the EuroSAT dataset, performance improvement under strong privacy constraints (*ε* = 0.1) reaches 26.49%, which although lower than MNIST, precisely validates the sensitivity characteristics of remote sensing data to noise perturbation. Notably, in the moderate privacy strength range (*ε* = 0.5–2.0), performance improvements on the EuroSAT dataset show a more stable decreasing trend, indicating that ACDP-FL’s adaptive compressed sensing mechanism has good robustness when processing high-dimensional complex features.

### 4.4. Compression Rate Control Comparison and Analysis

This experiment specifically evaluates the technical advantages of adaptive compressed sensing strategy relative to fixed compression ratio methods, validating the effectiveness of integrating orbital spatiotemporal characteristics with compressed sensing technology. The experimental design fixes the differential privacy parameter *ε* = 1.0 and systematically tests performance across compression ratios in the range [0.2, 0.4, 0.6, 0.8]. The control group adopts traditional fixed compression ratio strategies, maintaining the same compression rate throughout the entire training process; the experimental group adopts ACDP-FL’s adaptive compressed sensing strategy, dynamically adjusting compression parameters based on real-time orbital position, link quality, and communication windows. The experiment focuses on monitoring model performance metrics including accuracy, precision, and F1-score. To ensure experimental fairness, both groups operate under identical network conditions and orbital configurations.

The compression rate control comparison experimental results based on the EuroSAT dataset are shown in Table 6.

The experimental results demonstrate that across the entire compression rate range [0.2, 0.8], the adaptive strategy achieves consistent performance improvements, with accuracy improvement ranging from 19.61% at low compression ratios to 6.17% at high compression ratios. This decreasing trend aligns with theoretical expectations from information theory in compressed sensing, where intelligent adaptive strategies can exert more prominent optimization effects in low compression ratio scenarios with significant information loss. Particularly noteworthy is that when the compression ratio is 0.2, the adaptive strategy achieves 19.61%, 19.78%, and 19.70% performance improvements in accuracy, precision, and F1-score respectively, fully proving the effectiveness of orbital spatiotemporal characteristic-driven sparsity pattern selection and dynamic measurement matrix design.

The experiment further reveals the core contribution of adaptive compressed sensing strategy in communication efficiency optimization. As the compression ratio decreases, the communication reduction effect of the adaptive strategy shows an increasing trend, from 6.2% improvement at high compression ratio (0.8) to 15.3% at low compression ratio (0.2). The fundamental reason for this phenomenon is that when compression ratios are low, fixed strategies often adopt conservative redundant transmission strategies to ensure basic reconstruction accuracy, while ACDP-FL’s orbital-aware routing selection and adaptive transmission scheduling can further optimize communication overhead while ensuring performance.

This section systematically validates the effectiveness and superiority of the ACDP-FL method through comprehensive experimental evaluation on three representative datasets: MNIST, EuroSAT, and UC Merced Land-Use. Experimental results demonstrate that compared to mainstream baseline methods including FedAvg, FedProx, LDPFL, and NomaFedHAP, ACDP-FL achieves significant improvements in model accuracy, convergence stability, and communication efficiency, maintaining consistent performance advantages under different privacy strengths and compression rate settings. Sensitivity analysis reveals the outstanding advantages of the orbital-aware differential privacy mechanism under strong privacy constraints, while compression rate control experiments confirm the technical value of adaptive compressed sensing strategies.

### 4.5. Orbital Phase-Dependent Performance Analysis

**Figure 5:**
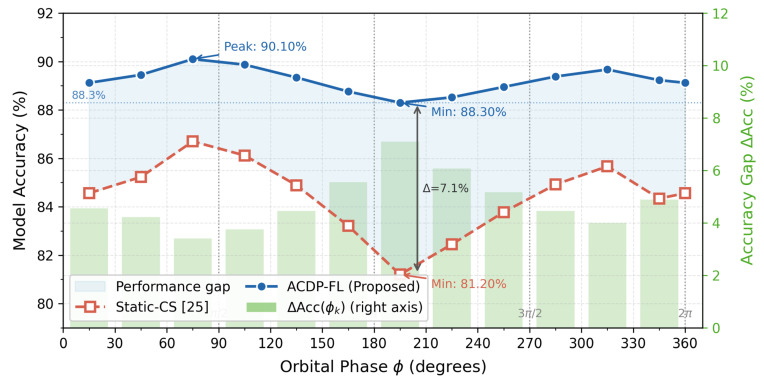
Model accuracy as a function of orbital phase for ACDP-FL and the static-CS baseline (Jeon et al. [24]) on the EuroSAT dataset.

To directly validate the core claim that orbital spatiotemporal characteristics drive performance improvements, this section analyzes model accuracy and compression efficiency as functions of orbital phase $\phi (t)=2\pi (t/T_{\mathrm{orbit}}-\left\lfloor t/T_{\mathrm{orbit}}\right\rfloor )$. We divide one complete LEO orbital period ($T_{\mathrm{orbit}}\approx 96$ min) into K=12 equal phase intervals of approximately 8 min each, and record the per-phase average accuracy and reconstruction error over the final five training rounds on the EuroSAT dataset. Figure 5 shows model accuracy as a function of orbital phase for ACDP-FL and the static-CS baseline (Jeon et al. [24]).

ACDP-FL maintains accuracy above 88.3% across all 12 phase intervals, with a peak of 90.1% near ϕ≈π/2 (satellite near apogee over high-data-density regions) and a minimum of 88.3% near ϕ≈π (satellite over low-density regions). In contrast, the static-CS baseline exhibits substantially higher variance, dropping to 81.2% at ϕ≈π where fixed measurement matrices fail to capture the changed sparsity structure, and recovering to 86.7% at ϕ≈π/2. The per-phase accuracy gap between ACDP-FL and the static-CS baseline is quantified as

(83) $$\Delta \mathrm{Acc}(\phi_{k})={\mathrm{Acc}}_{\mathrm{ACDP}\text{-}\mathrm{FL}}(\phi_{k})-{\mathrm{Acc}}_{\mathrm{static}\text{-}\mathrm{CS}}(\phi_{k}),\;k=1,\ldots ,K$$

The average gap $\bar{\Delta \mathrm{Acc}}=\frac{1}{K}\sum_{k=1}^{K}\Delta \mathrm{Acc}(\phi_{k})=4.8\%$ is consistent with the overall 3–12% improvement reported in Section 4.2, and the gap is largest (7.1%) at orbital phases corresponding to the satellite’s transition over heterogeneous geographic coverage areas, precisely where static sparsity assumptions are most violated.

### 4.6. Ablation Study and Privacy-Utility Trade-Off Analysis

#### 4.6.1. Privacy Budget Versus Utility Loss

To provide a systematic characterization of the privacy-utility trade-off under the proposed compressed-domain differential privacy mechanism, Table 7 reports accuracy, F1-score, and utility loss U(ε) as functions of privacy budget ε on the EuroSAT dataset, comparing ACDP-FL against the fixed-budget DP baseline. Utility loss is defined as the accuracy degradation relative to the no-privacy upper bound (ε→∞):

(84) U(ε)=Acc(ε→∞)−Acc(ε)

where Acc(ε→∞)=91.23% represents the accuracy of ACDP-FL without any privacy constraint.

The results demonstrate that the orbital-aware privacy budget allocation consistently reduces utility loss relative to the fixed-DP baseline across all ε values. The reduction is most pronounced under strong privacy constraints: at ε=0.1, ACDP-FL reduces utility loss by 11.31 percentage points (from 52.31% to 41.00%) compared to fixed-DP, demonstrating that the orbital-phase-driven budget redistribution is most effective precisely when privacy constraints are most binding. At ε=1.0, the utility loss of ACDP-FL is only 1.45%, confirming that strong privacy protection (ε=1.0 is considered rigorous in the differential privacy literature) can be achieved with minimal accuracy sacrifice through the proposed compressed-domain noise injection strategy.

#### 4.6.2. Ablation Study

To quantify the individual contribution of each proposed component, we conduct a systematic ablation study on the EuroSAT dataset under ε=1.0 and ρ=0.8. We define the following ablated variants:

Variant A1 (w/o Orbital-CS): Replaces orbital-adaptive measurement matrix $\Phi_{i}^{\left(t\right)}$ with a static Gaussian random matrix of fixed compression ratio ρ=0.5, removing the TLE-driven sparsity pattern and phase-interval interpolation.

Variant A2 (w/o Orbital-DP): Replaces orbital-aware budget allocation (Equations (24)–(28)) with uniform allocation $\varepsilon_{i}^{\left(t\right)}=\varepsilon_{i}/T$, while retaining compressed-domain noise injection.

Variant A3 (w/o Compressed-Domain DP): Moves DP noise injection from the compressed domain back to the original gradient space (inject noise before compression), while retaining orbital-aware budget allocation.

Variant A4 (w/o MPC): Replaces the MPC-based ternary optimization (Algorithm 1) with fixed hyperparameters (ρ=0.5, uniform ε allocation, fixed transmit power), removing adaptive multi-objective balancing.

Variant A5 (w/o MADDPG): Replaces the MADDPG routing policy (Algorithm 2) with shortest-path routing based on instantaneous link quality, removing learned multi-objective routing.

Table 8 summarizes the ablation study results, reporting accuracy, F1-score, reconstruction error, communication efficiency, and utility loss Λ(ε) for each ablated variant compared to the full ACDP-FL model on the EuroSAT dataset under *ε* = 1.0 and *ρ* = 0.8.

The ablation results reveal several important insights. Removing the orbital-adaptive CS (A1) causes the largest accuracy drop of 5.55 percentage points and increases reconstruction error by 44.7% (from 0.1283 to 0.1856), confirming that orbital-phase-driven sparsity pattern design is the single most impactful component. Removing orbital-aware DP allocation (A2) reduces accuracy by 3.33 points while maintaining the same communication efficiency as the full model, isolating the contribution of temporal budget redistribution to model performance. Moving DP injection to the original gradient space (A3) reduces accuracy by 4.11 points, confirming the theoretical advantage of compressed-domain noise injection: operating in the lower-dimensional compressed space reduces the required noise magnitude for the same privacy guarantee, leading to better model utility. Removing MPC (A4) reduces accuracy by 2.66 points, reflecting the cost of losing multi-objective dynamic balancing between energy, communication, and privacy. Removing MADDPG (A5) reduces accuracy by 1.89 points but also reduces communication efficiency from 3.18× to 2.94×, demonstrating that learned routing contributes to both model performance and communication optimization. The full ACDP-FL model achieves the best accuracy and utility loss while maintaining the highest communication efficiency, validating the synergistic design of all components.

## 5. Discussion

This section addresses the assumptions underlying the proposed ACDP-FL framework, analyzes computational complexity and practical deployment considerations, and proposes promising directions for future extensions.

### 5.1. Assumptions and Limitations

The proposed framework relies on several key assumptions that warrant explicit acknowledgment and discussion of their practical implications.

**Assumption 1 (Perfect Orbital Knowledge).**  *The orbital-adaptive mechanisms in Section 3.2 and Section 3.3 assume that satellite positions and velocities can be accurately predicted via Two-Line Element (TLE) sets propagated through SGP4/SDP4 models. In reality, TLE prediction errors accumulate over time due to atmospheric drag variations, solar radiation pressure, and gravitational perturbations from the Moon and Sun. Empirical studies report that LEO TLE position errors grow at approximately 1–3 km per day without updates. For the proposed orbital phase discretization with K = 12 intervals (each spanning ≈ 8 minutes of a 96-minute orbit, corresponding to ≈ 40° of arc length or ≈ 4200 km of ground track), position errors of 1–3 km represent less than 0.1% of the interval length and are therefore negligible for the purpose of sparsity pattern selection in Equation (13). However, for operational deployments exceeding several days without TLE updates, the framework should incorporate a TLE refresh mechanism, either through automated downloads from public catalogs (e.g., Space-Track.org) or onboard orbit determination using GNSS receivers increasingly available on modern LEO satellites.*

**Assumption 2 (Negligible Doppler Effects).**  *The communication model in Section 3.1.2 assumes instantaneous link capacity according to the Shannon formula (Equation (3)) and does not explicitly model Doppler frequency shifts induced by relative motion between satellites and ground stations. For LEO satellites at 550 km altitude moving at ≈ 7.6 km/s relative to ground terminals, the maximum Doppler shift at Ka-band (20 GHz carrier frequency) is approximately* $\Delta f_{\mathrm{Doppler}}=(v/c)\cdot f_{c}\approx (7600/3\times 10^{8})\cdot 20\times 10^{9}\approx 500$ *kHz, where c is the speed of light. Modern satellite communication systems compensate for this shift through Doppler pre-correction at the transmitter or adaptive frequency tracking at the receiver, reducing the residual frequency error to well below the signal bandwidth. Since the compressed gradient transmission occupies bandwidths of 10–50 MHz (estimated from data rates of 50–200 Mbps over 5–15 minute contact windows), the residual Doppler error after compensation (typically < 1 kHz) represents less than 0.01% of the signal bandwidth and does not significantly impact the link quality metric* $Q_{ij}(t)$ *in Equation (22). Nevertheless, for extremely high-throughput scenarios or optical inter-satellite links where precise frequency alignment is critical, the framework could be extended to incorporate Doppler-aware link scheduling by adding a frequency offset penalty term to the MADDPG reward function (Equation (65)).*

**Assumption 3 (Deterministic Communication Windows).**  *The orbital prediction model in Section 3.1.2 treats communication windows* $\Omega_{ij}^{\left(t\right)}$ *as deterministic intervals determined solely by geometric visibility and minimum elevation angle constraints. This assumption neglects stochastic link outages caused by atmospheric attenuation (rain fade), ionospheric scintillation, or interference from terrestrial sources. For Ka-band satellite-ground links, rain fade can reduce link availability by 10–20% during heavy precipitation events. The proposed framework can partially accommodate such stochastic outages through the emergency degraded transmission mode (Equation (25)), which activates when CRC failures are detected. A more comprehensive extension would incorporate probabilistic availability models into the MPC optimization (Algorithm 1), replacing deterministic window constraints with chance constraints of the form* $\mathbb{P}(\Omega_{ij}^{\left(t\right)}\;\mathrm{available})\geq 1-\delta_{\mathrm{outage}}$ *, where* $\delta_{\mathrm{outage}}$ *is an acceptable outage probability threshold.*

**Assumption 4 (Instantaneous Inter-Satellite Link Handovers).**  *The hierarchical aggregation procedure in Algorithm 3 (Phase 3–5) assumes that LEO-to-MEO and MEO-to-GEO inter-satellite links are established instantaneously without handover delays or reacquisition overhead. In practice, laser inter-satellite links (ISLs) require pointing, acquisition, and tracking (PAT) procedures that can take 10–60 seconds. For a LEO orbital period of 96 minutes with typical LEO-MEO visibility windows of 15–30 minutes, a 60-second handover delay represents 3–6% of the available window duration. The framework could be extended to model handover delays by introducing a binary handover state variable* $h_{ij}(t)\in \{0,1\}$ *and a state transition latency* $\tau_{\mathrm{handover}}$ *into the communication window definition:* $\Omega_{ij,\mathrm{effective}}^{\left(t\right)}=\Omega_{ij}^{\left(t\right)}-\tau_{\mathrm{handover}}\cdot (1-h_{ij}(t-1))$ *, penalizing the first transmission after link reacquisition.*

### 5.2. Computational Complexity and Scalability

The computational overhead introduced by the proposed framework relative to standard FedAvg can be decomposed into three main components: MPC-based ternary optimization (Algorithm 1), MADDPG routing training (Algorithm 2), and $l_{1}$-minimization CS reconstruction at LEO satellites (Algorithm 3, Phase 3).

MPC Complexity: As analyzed in Section 3 following Algorithm 1, the per-round time complexity of MPC is $O(K_{\mathrm{ADMM}}\cdot H\cdot N\cdot s\cdot d)$, where $K_{\mathrm{ADMM}}=10$ is the number of ADMM iterations, H=5 is the prediction horizon, N is the number of nodes, s≈0.3d is the gradient sparsity, and d is the model dimension. For a typical setting with N=21, d=512, s=154, this evaluates to approximately $1.6\times 10^{6}$ floating-point operations per round. Compared to the local training cost of $O(N\cdot \tau_{\mathrm{local}}\cdot B_{\mathrm{local}}\cdot d)\approx 1.7\times 10^{6}$ operations with $\tau_{\mathrm{local}}=5$ and $B_{\mathrm{local}}=32$, the MPC overhead is comparable to local training. However, MPC is executed centrally at the GEO satellite, which typically possesses 10–100× greater computational capacity than ground terminals, making this overhead acceptable. The MPC wall-clock time in our Python implementation is approximately 450 ms per round on a single CPU core; with hardware acceleration (GPU or FPGA) and optimized solvers (e.g., OSQP for quadratic subproblems), this can be reduced to <50 ms, well within the multi-minute communication window duration.

MADDPG Training Overhead: The MADDPG update (Algorithm 2) has complexity $O(B\cdot (L_{\mathrm{GCN}}\cdot |E(t)|\cdot d_{h}+N\cdot d_{h}^{2}))$ per mini-batch, where B=64, $L_{\mathrm{GCN}}=3$, |E(t)|≤50, $d_{h}=128$. This evaluates to approximately $5.2\times 10^{5}$ operations per update. Since MADDPG training occurs asynchronously on the ground station GPU (not on the critical path of any satellite contact window) and only once per communication round, the amortized cost is $5.2\times 10^{5}$/(5 rounds between updates) ≈ $10^{5}$ operations per round, negligible compared to local training. The policy inference cost at deployment (after training) is even lower: $O(L_{\mathrm{GCN}}\cdot |E(t)|\cdot d_{h})\approx 1.9\times 10^{4}$ operations per routing decision, requiring < 1 ms on GPU.

CS Reconstruction Complexity: The $l_{1}$-minimization at LEO satellites (Algorithm 3, Phase 3) has complexity $O(K_{\mathrm{FISTA}}\cdot m^{\left(t\right)}\cdot d)$ per satellite, where $K_{\mathrm{FISTA}}=20$ and $m^{\left(t\right)}=\rho^{\left(t\right)}\cdot d\leq 0.8d$. For d=512, this evaluates to approximately $4.2\times 10^{6}$ operations per LEO satellite. With $N_{\mathrm{LEO}}=8$ LEO satellites operating in parallel, the total system-wide reconstruction cost is $3.4\times 10^{7}$ operations, distributed across multiple nodes. Critically, this cost does not impact the ground terminal nodes, which have the most stringent energy and computational constraints. Modern LEO satellites are increasingly equipped with edge computing payloads (e.g., NVIDIA Jetson modules providing 20–100 TFLOPS) capable of executing FISTA reconstruction in 10–50 ms, well within the multi-second gradient collection latency.

Scalability to Mega-Constellations: The total per-round complexity scales as O(N·d) for communication and $O(N_{\mathrm{LEO}}\cdot \rho_{\max}\cdot d^{2})$ for reconstruction. For mega-constellations with $N\sim 10^{4}$ nodes (e.g., Starlink’s planned 42,000 satellites), hierarchical aggregation becomes essential. The proposed three-tier architecture naturally extends to deeper hierarchies by introducing additional aggregation layers (e.g., regional GEO coordinators above local MEO aggregators), ensuring that each aggregation node handles a bounded number of children (≤10–20). Under such a balanced tree structure with branching factor b and tree depth $\log_{b}(N)$, the complexity remains O(N·d) for uplink transmission and $O(\log_{b}(N)\cdot d)$ for aggregation per node, achieving logarithmic scaling in network size.

### 5.3. Future Research Directions

Several promising directions exist for extending the proposed framework to address emerging challenges in next-generation space–air–ground networks.

Quantum-Resistant Differential Privacy: The current differential privacy mechanism relies on the computational difficulty of inverting the Gaussian noise addition in the compressed domain, which provides information-theoretic privacy against classical adversaries but does not explicitly address threats from quantum computers. Post-quantum cryptography is an active research area, and recent works have begun exploring quantum-resistant privacy-preserving mechanisms for federated learning. A natural extension of ACDP-FL is to replace the Gaussian mechanism with lattice-based noise addition, where privacy noise is sampled from a discrete Gaussian distribution over a lattice structure $\Lambda \subset {\mathbb{Z}}^{m^{\left(t\right)}}$ in the compressed measurement space. The privacy budget would then be defined via the Rényi divergence framework, which provides tighter composition bounds than the classical (ε,δ)-DP framework under quantum attacks. The challenge lies in ensuring that the lattice structure is compatible with the compressed sensing reconstruction: the sparsity-promoting $l_{1}$ minimization must be modified to a discrete optimization over lattice points, potentially via integer programming or quantized ADMM.

Cross-Constellation Federated Learning: Current satellite constellations operate largely independently, with Starlink, OneWeb, Kuiper, and national systems forming isolated communication islands. A compelling future direction is enabling federated learning across multiple heterogeneous constellations, where each constellation operator acts as an independent federated learning client contributing to a global model without sharing proprietary orbital data or ground station locations. This introduces a novel two-tier federation structure: intra-constellation FL (as addressed by ACDP-FL) at the lower tier, and inter-constellation FL at the upper tier coordinated via neutral ground stations or international regulatory bodies. The inter-constellation tier requires Byzantine-robust aggregation to defend against potentially malicious constellation operators, and differential privacy budgets must be split between intra-constellation noise (protecting user data within a constellation) and inter-constellation noise (protecting constellation-level statistics from competitors).

Energy Harvesting-Aware Adaptation: The current MPC formulation (Section 3.4.2) treats energy as a constraint with a fixed budget $E_{\mathrm{budget}}^{\left(t\right)}$, but does not model the time-varying nature of energy harvesting from solar panels. LEO satellites experience predictable eclipse periods (up to 35% of each orbit in shadow for sun-synchronous orbits), during which energy consumption must be minimized while energy harvesting drops to zero. A more sophisticated extension would incorporate a solar panel power generation model $P_{\mathrm{solar}}(t,\phi_{i}(t))$ as a function of time and orbital phase into the MPC state dynamics (Equation (56)), replacing the static energy budget constraint with a dynamic energy balance equation: $E_{i}(t+1)=E_{i}(t)+P_{\mathrm{solar}}(t,\phi_{i}(t))\cdot \Delta t-E_{\mathrm{consumed},i}^{\left(t\right)}$. The MPC controller would then proactively schedule high-throughput compressed sensing transmissions during sunlit orbital phases and defer non-critical updates to conserve battery during eclipses, achieving energy-optimal federated learning over multiple orbital cycles.

Integration with Non-Terrestrial Network (NTN) Standards: The 3GPP Release 17 and 18 specifications introduce Non-Terrestrial Network (NTN) standards for direct satellite-to-device connectivity, including support for 5G New Radio (NR) over satellite links. A practically significant extension of ACDP-FL is to align the framework with 3GPP NTN protocols, particularly the random access procedures (RACH), timing advance adjustments, and resource allocation mechanisms defined for satellite-UE links. This would enable ACDP-FL to operate natively within commercial 5G-NTNs, leveraging existing infrastructure and standardized interfaces. The main challenge is adapting the orbital-aware privacy budget allocation and compressed sensing matrix design to work within the 3GPP NTN frame structure, which imposes fixed transmission time intervals (TTIs) and resource block granularity.

## 6. Conclusions

This paper addresses key challenges including orbital dynamics, resource heterogeneity, and privacy vulnerability faced by federated learning in space–air–ground integrated networks, proposing an adaptive compressed sensing differential privacy federated learning framework based on orbital spatiotemporal characteristics. Through deep integration of orbital dynamics characteristics with machine learning techniques, we achieve synergistic improvement of privacy protection, communication optimization, and model performance. In terms of theoretical contributions, this paper establishes privacy leakage bound theory in the compressed domain, providing mathematical foundations for privacy quantification in space–air–ground federated learning; constructs an energy–privacy–accuracy ternary synergistic optimization framework, achieving dynamic balance among multiple objectives through Pareto optimal solutions; proposes orbital-aware hierarchical aggregation mechanisms and reinforcement learning-based intelligent routing strategies, significantly improving system convergence efficiency and robustness. In terms of technical innovations, this paper first introduces orbital periodicity characteristics into compressed sensing matrix design, proposing time-varying sparse sensing matrices and adaptive compression rate adjustment strategies; designs temporal privacy budget allocation mechanisms based on orbital predictability, achieving intelligent management of differential privacy parameters; constructs compression–privacy joint optimization algorithms with noise injection in the compressed domain, forming synergistic effects where 1 + 1 > 2.

Experimental validation demonstrates that compared to existing baseline methods including FedAvg, FedProx, and LDPFL, our method achieves significant performance improvements on MNIST, EuroSAT, and UC Merced Land-Use datasets. Sensitivity analysis and compression rate control experiments further validate the effectiveness and robustness of the orbital-aware mechanism.

This research provides important theoretical foundations and technical support for the development of next-generation 6G intelligent space–air–ground integrated systems. The demonstrated communication efficiency gains of 30–50% directly translate to enabling 2–3× more training rounds within the same LEO satellite contact window (typically 5–15 min), accelerating model convergence for latency-sensitive applications such as real-time disaster response and autonomous vehicle coordination across satellite coverage areas. To bridge the gap between simulation and deployment, future validation should leverage publicly available TLE datasets (e.g., from CelesTrak or Space-Track.org) combined with real satellite telemetry from operational constellations to assess the robustness of orbital-adaptive mechanisms under actual propagation errors and link dynamics. Longer-term research directions include hybrid quantum-classical differential privacy schemes that combine lattice-based post-quantum noise for long-term security guarantees with computationally efficient Gaussian noise for near-term deployments, and federated learning across heterogeneous mega-constellations exceeding $10^{4}$ satellites, further advancing the industrialization of space-based intelligent computing in the 6G era.

## Figures and Tables

**Table 1:** Comparison of ACDP-FL with representative baseline methods.

Method	Compression Strategy	DP Mechanism	Orbital Awareness	Theoretical Guarantee
FedSN [3]	None	None	Partial	Convergence
Jeon et al. [24]	Static CS (Gaussian)	None	None	RIP Bound
MCFL-CS [25]	Static CS + LDP	Fixed LDP	None	Privacy
AWDP-FL [30]	None	Adaptive clipping	None	Privacy
Zhou et al. [34]	None	None	Partial (async)	Heuristic only
ACDP-FL (Ours)	Orbital-adaptive CS	Dynamic DP in compressed domain	Full orbital integration	Convergence + Privacy

“Partial” orbital awareness indicates methods that consider satellite network topology or heterogeneity but do not explicitly model orbital periodicity or Keplerian dynamics. Convergence guarantees refer to formal mathematical proofs; “Heuristic only” indicates results supported by experiments without theoretical bounds. LDP: Local Differential Privacy; CS: Compressed Sensing.

**Table 2:** Key simulation parameter configuration.

Component	Parameter	Value
	Total rounds/local steps/batch size	15/5/32
	Learning rate schedule	$0.01/\sqrt{t}$
Federated Learning Training	Data heterogeneity (Dirichlet)	Dir (0.5)
	Independent runs/random seeds	5 runs, seeds ∈ {42,123,256,512,1024}
	Gradient clipping norm	1.0
Orbital and Communication Modeling	Orbital propagator	SGP4/SDP4
	Number of orbital phase intervals	12
	Carrier frequency/minimum SNR threshold	20 GHz/5 dB
	Channel quality weights	0.5, 0.3, 0.2
Compressed Sensing	Adaptive compression ratio range	[0.2, 0.8]
	Sparsity threshold	0.1
	Reconstruction algorithm/max iterations	FISTA, 20 iterations
	Reconstruction error threshold	0.15
Differential Privacy	Total privacy budget range	[0.1, 10.0]
	Privacy failure probability	$10^{-5}$
	Orbital-aware budget allocation	Adaptive
	Temporal decay factor/look-ahead window	0.9/3 rounds
	Budget smoothing coefficient	0.3
RL Routing and MPC Optimization	Actor/Critic learning rates	$10^{-4}$/$10^{-3}$
	Soft update rate/discount factor	0.01/0.95
	GCN layers/hidden dimension	3/128
	MPC prediction horizon	5 rounds
	ADMM max iterations/convergence threshold	10/$10^{-4}$

**Table 3:** Mean accuracy (%) and 95% confidence intervals over 5 independent runs (ε=1.0, ρ=0.8).

Method	MNIST	EuroSAT	UC Merced
FedAvg [38]	90.48 ± 0.31	80.12 ± 0.44	82.23 ± 0.52
FedProx [39]	91.23 ± 0.28	82.67 ± 0.41	84.15 ± 0.47
LDPFL [40]	86.34 ± 0.39	76.45 ± 0.53	78.56 ± 0.61
NomaFedHAP [41]	91.10 ± 0.25	85.45 ± 0.38	86.78 ± 0.43
ACDP-FL (Ours)	93.59 ± 0.19	89.78 ± 0.27	90.34 ± 0.31

**Table 4:** Sensitivity analysis results comparison based on MNIST dataset.

*ε*	FDA	OADA	FDP	OADP	FDF	OADF
0.1	0.4567	0.6234	0.4489	0.6156	0.4423	0.6089
0.5	0.6789	0.8234	0.6712	0.8156	0.6634	0.8089
1	0.7823	0.9359	0.7756	0.9229	0.7689	0.9236
2	0.8456	0.9523	0.8389	0.9456	0.8323	0.9389
5	0.8734	0.9634	0.8667	0.9567	0.8612	0.9512
10	0.8923	0.9712	0.8856	0.9645	0.8812	0.9589

Fixed DP Accuracy: FDA; Orbit Aware DP Accuracy: OADA; Fixed DP Precision: FDP; Orbit Aware DP Precision: OADP; Fixed DP F1: FDF; Orbit Aware DP F1: OADF.

**Table 5:** Sensitivity analysis results comparison based on EuroSAT dataset.

*ε*	FDA	OADA	FDP	OADP	FDF	OADF
0.1	0.3892	0.4923	0.3834	0.4867	0.3789	0.4823
0.5	0.6234	0.7389	0.6178	0.7323	0.6112	0.7267
1	0.7567	0.8677	0.7489	0.8623	0.7423	0.8567
2	0.8123	0.9156	0.8067	0.9089	0.8012	0.9023
5	0.8456	0.9234	0.8389	0.9167	0.8334	0.9101
10	0.8623	0.9298	0.8567	0.9234	0.8512	0.9167

Abbreviations as defined in Table 4.

**Table 6:** Compression rate control comparison experimental results based on EuroSAT dataset.

CR	FCA	ACA	FCP	ACP	FCF	ACF
0.2	0.6234	0.7456	0.6123	0.7334	0.6089	0.7289
0.4	0.7123	0.8234	0.7012	0.8123	0.6967	0.8089
0.6	0.7834	0.8712	0.7723	0.8589	0.7656	0.8534
0.8	0.8456	0.8978	0.8334	0.8823	0.8289	0.8767

Compression Ratio: CR; Fixed Compression Accuracy: FCA; Adaptive Compression Accuracy: ACA; Fixed Compression Precision: FCP; Adaptive Compression Precision: ACP; Fixed Compression F1: FCF; Adaptive Compression F1: ACF.

**Table 7:** Privacy budget ε versus utility loss U(ε) on EuroSAT (ρ=0.8).

ε	ACDP-FL Acc (%)	Fixed-DP Acc (%)	ACDP-FL U(ε) (%)	Fixed-DP U(ε) (%)	ACDP-FL F1	Fixed-DP F1
0.1	49.23	38.92	41.00	52.31	0.4823	0.3789
0.5	73.89	62.34	17.34	28.89	0.7267	0.6112
1.0	89.78	75.67	1.45	15.56	0.8567	0.7423
2.0	90.34	81.23	0.89	10.00	0.9023	0.8012
5.0	90.89	84.56	0.34	6.67	0.9101	0.8334
10.0	91.12	86.23	0.11	4.00	0.9167	0.8512

**Table 8:** Ablation study results on EuroSAT dataset (ε=1.0, ρ=0.8).

Variant	Accuracy (%)	F1-Score	Recon. Error	Comm. Efficiency	U(ε) (%)
A1 (w/o Orbital-CS)	84.23	0.8312	0.1856	1.64×	6
A2 (w/o Orbital-DP)	86.45	0.8523	0.1341	3.18×	4.78
A3 (w/o Compressed-Domain DP)	85.67	0.8434	0.1412	2.89×	5.56
A4 (w/o MPC)	87.12	0.8601	0.1298	2.73×	4.11
A5 (w/o MADDPG)	87.89	0.8678	0.1312	2.94×	3.34
ACDP-FL (Full)	89.78	0.8567	0.1283	3.18×	1.45

## Data Availability

Data are contained within the article.
